# p140Cap modulates the mevalonate pathway decreasing cell migration and enhancing drug sensitivity in breast cancer cells

**DOI:** 10.1038/s41419-023-06357-z

**Published:** 2023-12-20

**Authors:** Giorgia Centonze, Dora Natalini, Silvia Grasso, Alessandro Morellato, Vincenzo Salemme, Alessio Piccolantonio, Giacomo D’Attanasio, Aurora Savino, Olga Teresa Bianciotto, Matteo Fragomeni, Andrea Scavuzzo, Matteo Poncina, Francesca Nigrelli, Mario De Gregorio, Valeria Poli, Pietro Arina, Daniela Taverna, Joanna Kopecka, Sirio Dupont, Emilia Turco, Chiara Riganti, Paola Defilippi

**Affiliations:** 1 Department of Molecular Biotechnology and Health Sciences, Molecular Biotechnology Center, University of Torino, Via Nizza 52, 10126 Torino, Italy; 2 UCL, Bloomsbury Institute of Intensive Care Medicine, Division of Medicine, University College London, WC1E 6BT London, UK; 3 Department of Oncology, University of Torino, Italy; Molecular Biotechnology Center, Piazza Nizza 44, 10126, Torino, Italy; 4 Department of Molecular Medicine (DMM), University of Padova, Padua, Italy

**Keywords:** Cancer metabolism, Breast cancer

## Abstract

p140Cap is an adaptor protein involved in assembling multi-protein complexes regulating several cellular processes. p140Cap acts as a tumor suppressor in breast cancer (BC) and neuroblastoma patients, where its expression correlates with a better prognosis. The role of p140Cap in tumor metabolism remains largely unknown. Here we study the role of p140Cap in the modulation of the mevalonate (MVA) pathway in BC cells. The MVA pathway is responsible for the biosynthesis of cholesterol and non-sterol isoprenoids and is often deregulated in cancer. We found that both in vitro and in vivo, p140Cap cells and tumors show an increased flux through the MVA pathway by positively regulating the pace-maker enzyme of the MVA pathway, the 3-hydroxy-3-methyl-glutaryl-coenzyme A reductase (HMGCR), via transcriptional and post-translational mechanisms. The higher cholesterol synthesis is paralleled with enhanced cholesterol efflux. Moreover, p140Cap promotes increased cholesterol localization in the plasma membrane and reduces lipid rafts-associated Rac1 signalling, impairing cell membrane fluidity and cell migration in a cholesterol-dependent manner. Finally, p140Cap BC cells exhibit decreased cell viability upon treatments with statins, alone or in combination with chemotherapeutic at low concentrations in a synergistic manner. Overall, our data highlight a new perspective point on tumor suppression in BC by establishing a previously uncharacterized role of the MVA pathway in p140Cap expressing tumors, thus paving the way to the use of p140Cap as a potent biomarker to stratify patients for better tuning therapeutic options.

## Introduction

The Mevalonate (MVA) pathway is responsible for the synthesis of cholesterol and non-sterol isoprenoids such as geranylgeranyl-diphosphate (GGPP) and farnesyl-diphosphate (FPP), essential for protein prenylation, and ubiquinone (UQ) synthesis [1, 2]. The 3-hydroxy-3-methyl-glutaryl-coenzyme A reductase (HMGCR) catalyzes the rate-limiting step of the MVA pathway, and it is finely controlled via transcriptional and post-translational mechanisms to preserve adequate levels of intracellular cholesterol. For instance, in conditions of low intracellular cholesterol, the transcription factor sterol regulatory element (SRE)-binding protein 2 (SREBP2) is cleaved into the Golgi, and translocated to the nucleus, where it binds to the SREs in the promoters of HMGCR and other MVA pathway genes, activating their expression [3]. Moreover, HMGCR protein level and activity are strictly regulated according to the cellular cholesterol needs by ubiquitin-dependent proteasomal degradation and various post-translational modifications, such as the phosphorylation on Serine 872 by the AMP-activated protein kinase (AMPK) [4].

In cancer cells, cholesterol homeostasis is often altered, displaying an upregulation of the MVA pathway [5, 6]; however, cholesterol’s role in tumors is still under debate [7, 8]. For instance, the profile of the mutational status and expression levels of all the genes in diverse cancers made by The Cancer Genome Atlas (TCGA) project includes those involved in cholesterol metabolism, providing correlative support for a role of the cholesterol pathway in cancer [7, 8]. It has been well established that cholesterol metabolism is often influenced by the alteration of oncogenes and tumor suppressors in BC, such as PI3K and p53 [9, 10]. Indeed, cholesterol is an important metabolite to sustain cell proliferation; however, low cholesterol levels in the plasma membrane contribute to increased membrane fluidity, favouring tumor cell migration and motility [5]. HMGCR is the well-known target of the cholesterol-lowering drugs statins, which have been shown to exert antiproliferative effects on several types of BC cells, including triple-negative and Her2-positive subtypes [11, 12]. Statins are currently under clinical investigation for BC prevention and treatment since they are associated with increased survival and lowered recurrence in BC patients [5, 13]. For instance, several studies in BC patients demonstrate that the use of statins is associated with lower risk of BC-related deaths, independently whether statins were used pre- or post-diagnosis [14–19].

The p140Cap (p130Cas-associated protein) adaptor is encoded by the *SRCIN1* gene and acts as a scaffold for the formation of multi-protein complexes by interacting with several partners. Indeed, p140Cap shares some features with the “Intrinsic Disorder Proteins”; in this context, disordered regions of p140Cap could allow interactions with alternative binding partners to promote specific interactions among proteins [20–22]. It is physiologically expressed in neurons, performing critical functions in synaptic plasticity and memory formation [23, 24] and in epithelial tissues of mammary glands, lungs, colon, and kidneys. We have previously described that p140Cap has tumor suppressor roles in BC [25–27], colon cancer [28], and neuroblastoma [29]. In Her2-amplified BC and neuroblastoma patients, a p140Cap-positive status is associated with a significantly lower probability of developing a distant event and a clear difference in survival [27, 29]. In particular, we have found that p140Cap exerts a negative control on in vivo tumor growth and metastasis formation in Luminal A [25], Her2-amplified [27], and in triple-negative BC cell models [25, 26]. Indeed, p140Cap is able to reduce cancer cells aggressiveness through specific molecular mechanisms, such as the down-regulation of Src kinase activity [25], and the Rac-Tiam1 axis [5, 22, 27], or by inhibiting the β-Catenin activity in cancer stem cells and modulating the immune tumor microenvironment [30, 31]. We have previously described the interactome of p140Cap in BC, in which proteins belonging to the Metabolism category in the KEGG functional division, in general, are well-represented [32]. However, the role of p140Cap in the metabolism of BC cells has never been investigated.

Here, we describe p140Cap as a crucial regulator of cholesterol metabolism in BC cells. We demonstrate that p140Cap upregulates the MVA pathway by inducing HMGCR transcription via SREBP2 activation and reducing HMGCR E3 ligase-ubiquitin-dependent degradation. Moreover, p140Cap promotes cholesterol export and favours its accumulation in the plasma membrane, reducing membrane fluidity and cell migration. Finally, p140Cap expression enhances the sensitivity of BC cells to statins and to the combined treatment with conventional chemotherapy drugs and statins.

## Materials and methods

### Gene ontology analysis

Gene ontology (GO) analysis was performed for up-regulated genes in MDA-MB-231 p140Cap cells, obtained as described in [26]. The lists of genes were loaded on DAVID tool for functional annotation analysis (http://david.abcc.ncifcrf.gov/summary.jsp), using the Agilent Whole Genome Oligo Microarray 8x60K as background for the upregulated genes.

### Cell lines and antibodies

Culture media were from Invitrogen (Carlsbad, CA, USA). Fetal Calf serum (FCS) was from EuroClone (Pero, Milano, Italy). MDA-MB-231, SKBR3, HEK293T, 4T1, HeLa and MCF7 cell lines were obtained from ATCC (LGC Standards S.r.l. Italy Office, Italy). SKBR3 cells were cultured in McCoy’s medium 15% FCS. MDA-MB-231, HEK293T and HeLa cells were cultured in DMEM 10% FCS. NeuT-TUBO cells (TUBO) were derived from a spontaneous breast tumor in female BALB/c-MMTV-NeuT mice as described in [33] and cultured in DMEM 20% FCS. 4T1 cells were cultured in RPMI medium 10% FCS. MCF7 were cultured in MEM 10% FCS 0,1% insulin. Cells were maintained in media supplemented with penicillin/streptomycin.

p140Cap mouse monoclonal antibody was produced in our laboratory as previously described [27]. For Western Blot (WB) analysis, the following antibodies were used: GAPDH (MAB374, Millipore Corp. USA), HMGCR (sc-271595, Santa Cruz Biotechnology, Santa Cruz, CA), anti-phosphoserine antibody (Sigma Chemical Co.), calreticulin (ABR Affinity Bioreagents, Thermo Fisher Scientific, Waltham, MA), mono-polyubiquitin antibody (Axxora, Lausanne, Switzerland), anti Flotillin-1 (sc-25506, Santa Cruz Biotechnology, Santa Cruz, CA), anti α-transferrin receptor-1 (CD71) (Thermo Fisher Scientific), anti RAC1 (from Rac1 activation assay, Cell Biolabs, Inc., San Diego, CA), anti Protein Disulfide Isomerases (PDI) (Abcam ab2792), anti E-cadherin Monoclonal Antibody (HECD-1, 13-1700 Thermo Fisher Scientific), rabbit anti-RTN3 (a kind gift of Prof. Sara Sigismund) directed against amino acids 1-47, common to all isoforms [34]; anti GM130 (6110823, BD Transduction Laboratories, Franklin Lakes, NY). Secondary antibodies conjugated with peroxidase were purchased from GE Healthcare.

### Retroviral infection and transfections

To overexpress p140Cap in SKBR3, MDA-MB-231, 4T1, and TUBO cells, p140Cap cDNA was cloned into pBabe-puro vector. The retroviruses particles were produced by the calcium phosphate transfection of Platinum Retroviral Packaging Cell Lines (Cell BioLabs). 48 h after transfection, retrovirus particles were collected from the supernatant, filtered, and added to subconfluent cells. After 48 h, cells were washed and selected with 1 mg/ml puromycin (Sigma). The efficiency of infection was assessed by WB analysis. For SKBR3, TUBO, 4T1, and MDA-MB-231 cells, individual clones were isolated 20 days after the start of the selection. Four individual positive clones were pooled together to rule out clonal artifacts. Control cells (mock cells) were generated by using the empty vector.

p140Cap cDNA was cloned into the BamHI site of pcDNA3.1/Myc-Hys expression vector (Invitrogen) or in pEGFP N1 upon PstI enzyme digestion. HEK293T cells were transfected by calcium phosphate precipitation; 15 μg of cDNA was used to transfect one 10 cm plate, and cell density was 50–80% confluent on the day of transfection. cDNA was mixed with sterilized Milli-Q water and 50 µl of CaCl_2_ 2.5 M to a final volume of 500 µl. The mix was slowly dropwise added to 500 µl of HEPES buffered saline (HBS) by gentle vortexing. 48 h after transfection, cells were harvested for further analysis.

Transient transfections of ON-TARGET plus human SRCIN1 small-interfering RNA (siRNA) or ON-TARGET plus non-targeting siRNA (Dharmacon RNAi, GE Healthcare, Buckinghamshire, UK) were performed with Lipofectamine 2000 (Invitrogen, USA) according to manufacturer’s protocol. This patented approach is the best strategy to prevent off-target effects caused by both the sense and antisense strands while maintaining high silencing potency. Briefly, cells were plated on a six-well plate and transfected at 80% confluency. Either 5 ml of 20 µM p140Cap siRNA or non-targeting siRNA were added to each well, and cells were incubated for 48 h at 37 °C in a humidifier.

### MVA pathway metabolic flux

The de novo synthesis of cholesterol, GGPP, and UQ was measured by radiolabelling 5 × 10^6^ cells (after overnight starvation) with 1 µCi [^3^H] acetate (3600 mCi/mmol; Amersham Bioscience, Piscataway, NJ), for 24 h. Cells were washed twice with PBS and scraped in 200 µl PBS. Methanol (0.5 ml) and hexane (1 ml) were added to the cell suspension, which was stirred at room temperature for 1 h and centrifuged at 2000 g for 5 min. The upper phase containing hexane was transferred to a new test tube, and the lower phase was supplemented with 1 ml of hexane and stirred overnight. After a 5 min centrifugation at 2000 g, the upper phase was added to the previous one, and the solvent was allowed to evaporate at room temperature for 24 h. Cellular lipid extracts produced by this separation were re-suspended in 30 µl chloroform and then subjected to thin layer chromatography (TLC), using a 1:1 (v/v) ether/hexane solution as mobile phase. Each sample was spotted on pre-coated LK6D Whatman silica gels (Merck, Darmstadt, Germany) and allowed to run for 30 min. Solutions of 10 µg/ml cholesterol, GGPP, and ubiquinone were used as standards. The plates were exposed for 1 h to an iodine-saturated atmosphere, and the migrated spots were cut out. Their radioactivity was measured by liquid scintillation, using a Tri-Carb Liquid Scintillation Analyzer (PerkinElmer, Waltham, MA). Cholesterol, GGPP, and ubiquinone synthesis were expressed as fmoles/10^6^ cells, according to previously prepared titration curves. When MVA pathway inhibitors simvastatin [35–37], zoledronic acid [38–40], and squalestatin [41, 42] were used, cells were treated for 24 h before incubation with [^3^H] acetate. The SP1 PF429242 inhibitor (Sigma SML0667) that interferes with SREBP2 cleavage at the Golgi has been used at a concentration of 10 μM [43] for 24 h.

### HMGCR immunoprecipitation and activity

10 × 10^6^ cells (after overnight starvation) were rinsed with the lysis buffer (10 mM Tris, 100 mM NaCl, 20 mM KH_2_PO_4_, 30 mM EDTA, 1 mM EGTA, 250 mM sucrose, pH 7.5) supplemented with protease inhibitor cocktail set III (100 mM AEBSF, 80 mM aprotinin, 5 mM bestatin, 1.5 mM E-64, 2 mM leupeptin and 1 mM pepstatin; Merck), 1 mM Na_3_VO_4_, 1 mM NaF, 1 mM PMSF, 10 mM aprotinin and 10 mM DTT. After sonication (two bursts of 10 s; Labsonic sonicator, Sartorius Stedim Biotech S.A., Aubagne Cedex, France), cell lysates were centrifuged at 13000 g for 15 min at 4 °C. The supernatants were centrifuged at 100000 g for 1 h at 4 °C, using an Optima L-90K Beckman Coulter Ultracentrifuge (Beckman Coulter Inc, Fullerton, CA) to collect the microsomal fraction, which was re-suspended in 250 µl lysis buffer and stored at -80 °C until the use. To measure HMGCR expression, 50 µg microsomal extracts were immunoprecipitated with an anti-HMGCR antibody using 25 µL of PureProteome Magnetic Beads (Millipore, Bedford, MA) in the presence of 100 mM DTT and 1 mM mevalonic acid. Western blotting was performed as described above. To detect ubiquitinated and serine-phosphorylated HMGCR, immunoprecipitated HMGCR was probed with an anti-mono/polyubiquitin antibody and a biotin-conjugated anti-phosphoserine antibody, respectively. 10 µg microsomal proteins were probed with CRT antibody as a control of equal loading.

For the HMGCR activity assay, microsomal protein extracts were resuspended in lysis buffer (12.5 µg proteins in 25 µl), supplemented with 10 mM DTT, 5 mM NADP, and a NADPH-generating system (1.3 mM glucose 6-phosphate, 0.67 U/ml glucose-6 phosphate dehydrogenase, 33 mM MgCl_2_). The reaction was started by adding 60 nCi [^14^C] HMG-CoA (50–62 mCi/mmol, Amersham Bioscience). After a 20-min incubation at 37 °C, the reaction was stopped with 25 µl 10 N HCl. The samples were stirred for 30 min at 37 °C to ensure complete lactonization of mevalonic acid, centrifuged at 13000 g for 2 min, and separated by TLC on silica gel plates with hexane/acetone (1:1) as mobile phase. A 1 mM solution of purified mevalonolactone was used as standard. The labeled product, [^14^C] mevalonolactone, was recovered from the TLC plates and quantified by liquid scintillation. HMGCR activity was expressed as nmol [^14^C] mevalonolactone/mg cell proteins, according to a titration curve previously set.

### HMGCR ubiquitination assay

To measure the ubiquitination of HMGCR, microsomal compartments were isolated from cell as described above. The ubiquitination assay was performed on 100 μg of microsomal proteins, diluted in 100 μl of ubiquitination assay buffer (1 M Tris/HCl, 500 mM MgCl_2_, 10 mM DTT, pH 8), using the E3Lite Customizable Ubiquitin Ligase kit (LifeSensors Inc., Malvern, PA), and divided into 3 aliquots. The first aliquot was incubated for 30 min at 37 °C, in the presence of 5 nM E1 activating enzyme provided by the kit, 100 nM E2 conjugating enzyme Ube2g2 (LifeSensors Inc.), 200 μM ATP, 6 mM human recombinant ubiquitin, to monitor the physiological ubiquitination of HMGCR in mock and p140Cap cells. The second and the third aliquot were incubated in the same conditions, plus a saturating amount (1.5 μM) of human recombinant E3 ligases Trc8/RNF-139 (Abnova) or gp78/AMFR (Abnova), to dissect the role of these E3 ligases, known to ubiquitinate HMGCR [44]. After two washing steps in PBS-Tween 0.1% v/v containing 5% w/v BSA, HMGCR was immunoprecipitated as detailed above, then incubated with the biotinylated anti-ubiquitin antibody of the kit, followed by the streptavidin/horseradish peroxidase-conjugated polymer and enhanced chemiluminescence detection reagent. The chemiluminescent signal was read using a Synergy HT Multi-Detection Microplate Reader. A blank was performed in the absence of microsomal extracts and its luminescence was subtracted from the luminescence of each sample. The results were expressed as relative luminescence units (RLU)/mg of microsomal proteins.

### Immunoblotting

Cells were lysed using a RIPA buffer (50 mM Tris (Ph 7.5), 150 mM NaCl, 1% TritonX-100, 1% Na Deoxycholate, 0.1% SDS, and protease inhibitors). Cell lysates were centrifuged at 13000 g for 15 min, and supernatants were collected and assayed for protein concentration using the Bio-Rad protein assay method (Biorad, Hercules, CA, USA). Proteins were run on SDS–PAGE under reducing conditions, transferred to PVDF membranes, incubated with specific antibodies, and detected with peroxidase-conjugated secondary antibodies and the chemiluminescent ECL reagent.

### In vivo tumor growth

Six/eight-week-old female BALB/c mice were purchased from Charles River Laboratories (Calco, Italy) and treated in accordance with the European Community guidelines. 10^4^ 4T1 and 10^5^ TUBO cells were suspended in 50 μl of PBS and then injected into the left fat pad of BALB/c mice. Tumor size was evaluated every two days using a caliper in blind experiments: maximum and minimum diameter were measured, and the volume was calculated using the following ellipsoid formula: (4/3π(d/2) ^ 2 * D/2). Mice were euthanized using a CO_2_ chamber when the tumor was approximately 500 mm^3^.

### Tumors metabolic analysis

Tumors were homogenized in 750 µL PBS using the Tissue Lyser II device (Qiagen), as per manufacturer’s instructions, then sonicated (two bursts of 10 s; Labsonic sonicator, Sartorius Stedim Biotech S.A., Aubagne Cedex, France). 500 µL were centrifuged at 13000 g for 15 min at 4 °C. The supernatants were centrifuged at 100000 g for 1 h at 4 °C, using a Optima L-90K Beckman Coulter Ultracentrifuge (Beckman Coulter Inc, Fullerton, CA) in lysis buffer (10 mM Tris, 100 mM NaCl, 20 mM KH_2_PO_4_, 30 mM EDTA, 1 mM EGTA, 250 mM sucrose, pH 7.5) supplemented with protease inhibitor cocktail set III, 1 mM Na_3_VO_4_, 1 mM NaF, 1 mM4-(2-aminoethyl)benzenesulfonyl fluoride (PMSF), 10 mM aprotinin and 10 mM dithiothreitol (DTT). The pellet (corresponding to microsomal fraction) was re-suspended in 500 µl lysis buffer and stored at −80 °C until use. 250 µl was used for the measurement of HMGCR activity, and 250 µL of tumor homogenates before the ultracentrifugation was used to measure total cholesterol. For both total and membrane-associated cholesterol, we used the Cholesterol Fluorimetric Assay kit (Cayman Chemical, Ann Arbor, MI) as per the manufacturer’s instructions. Results were expressed as µmol cholesterol/mg cell proteins.

### qRT–PCR

Total RNA was extracted using the RNeasy Mini kit (Qiagen, CA) with DNase I treatment. RT–PCR was performed on 1 μg total RNA with the High-Capacity cDNA Reverse Transcription kit from Applied Biosystems (Thermo Fisher Scientific). Gene expression was assessed by quantitative real-time PCR with the GeneAmp 7500 system and Platinum® SYBR® Green qPCR SuperMix-UDG Products (Invitrogen Life Science Technologies). Each sample was tested in triplicate. The ΔΔ-Ct method was used to calculate relative fold-changes normalized against RPL32. The primers used were the following:

HMGCR (F: CCCCTCTCCAGGTGTTCACA; R: AATTGAGGTAGGTTTCATAGAGATGCT); SREBF-2 (F: CGAATTGAAAGACCTGGTCATG; R: TCCTCAGAACGCCAGACTTGT); SQLE (F: CGTGCTCCTCTTGGTACCTCAT; R: CGGTCAAGGCGGAGATTATC); CYP51A1 (F: TGCAGCCTGGCTCTTACCA; R: AGCTCTGTCCCTGCGTCTGA); RPL32A (R: TGTGAGCGATCTCGGCAC; F: TTCCTGGTCCACAACAACGTCAAG); HMGCS1 (F: GGGCAGGGCATTATTAGGCTAT; R: TTAGGTTGTCAGCCTCTATGTTGAA); MVK (F: TGGACCTCAGCTTACCCAACA; R: GACTGAAGCCTGGCCACATC); PMVK (F: CCGCGTGTCTCACCCTTT; R: GACCGTGCCCTCAGCTCAT); MVD (F: TGAACTCCGCGTGCTCATC; R: CGGTACTGCCTGTCAGCTTCT); IDI1 (F: TTTCCAGGTTGTTTTACGAATACG; R: TCCTCAAGCTCGGCTGGAT); FDPS (F: CTTCCTATAGCTGCAGCCATGTAC; R: GCATTGGCGTGCTCCTTCT); FDFT1 (F: TCAGACCAGTCGCAGTTTCG; R: CTGCGTTGCGCATTTCC); LSS (F: TGCAGAAGGCTCATGAGTTCCT; R: TCTGGTAGTCGGGAGGGTTATC); TM7SF2 (F: GCCACCCTCACCGCTTT; R: GCTACCTGCGCCTTCATGTAG); SC4MOL (F: GAAAAGCCGGCACCAAGA; R: TCAAAGAGAGAATCAGCTCAAACTG); NSDHL (F: AGAATCAGGCCAAGAGATGCA; R: TGTGCTGCCCCAGGAATC); DHCR7 (F: GGCATCCCAGCTCCAACTC; R: GGGCTCTCTCCAGTTTACAGATGA); DHCR24 (F: CAAGTACGGCCTGTTCCAACA; R: CGCACAAAGCTGCCATCA).

### Luciferase assay

The pLDLR-Luc plasmid (Addgene #14940) harboring the SREBP-responsive Sterol Regulatory Element (SRE) sequence (ATCACCCCAC) and the pLDLR-Luc mutSRE construct (Addgene #14945) containing the SREBP-unresponsive mutant SRE (ATAACCCCAC) were gifts from Giannino del Sal. pLDLR-Luc (208 ng/cm2) or pLDLR-Luc mutSRE (208 ng/cm2) plasmids were co-transfected with CMV-Renillia (93 ng/cm2) and p140Cap-MYC (208 ng/cm2) plasmids in HEK293T cells by using the calcium phosphate transfection method. MYC (125 ng/cm2) construct instead of p140Cap-MYC was used as a negative control. 24 h after transfection, the Firefly/Renilla signal was analyzed in cell lysates using the Dual-Luciferase Reporter Assay System (Promega E1910). The experiment was performed in three biological replicates and four technical replicates, each in a 96-well plate.

### Nuclear and cytoplasmic extraction

HEK293T cells were transfected with p140Cap vector or the empty vector. Subsequently two platelets of cells for each condition were frozen at 6, 24, 30 and 48 h after transfection. Then 400 μl of Hypotonic isolation buffer (HIB), 10 mM HEPES (pH = 7.4) were added in each plate and left on the rocker for 20 min at 4 °C. Cells lysate was collected and homogenised through a 22 g needle syringe for ten times before to be centrifuged at 2500 rpm for 5 min. After the centrifugation, the cytosolic fraction present in the supernatant was collected and quantified by Bradford’s method, while the nuclear fraction consisting in the pellet was washed twice with 600 μl of HlB buffer and resuspended in 300 μl of SDS 1% lysis buffer and vortexed at 95 °C for 25 min before being quantified by Lowry’s method. The same amount of protein extract from each fraction was then loaded in SDS-page and analysed by WB.

### p140Cap localization in ER by immunofluorescence

HeLa cells were transfected with p140Cap-RFP and GFP-C1-PLCdelta-PH (Addgene plasmid #21179, Kind gift of Tobias Meyer) or ESYT1-GFP (Addgene plasmid # 66830, kind gift of Prof. S. Sigismund) constructs and fixed in PBS with 4% Paraformaldehyde + 4% sucrose, 10 min at RT cells. Permeabilization was performed with PBS 0,1% TritonX100, Sigma, for 5 min, then the cells were incubated for 30 min with blocking solution (PBS, 5% BSA, Sigma and 10% Goat Serum) and with primary antibody for GM130 for 1 h at room temperature in PBS, 1% BSA, 2% Goat Serum. AlexaFluor-488 secondary antibodies were added for 1 h protected from light. Samples were rinsed three times in PBS and mounted on glass slides with ProLong (Thermo Fisher Scientific) mounting medium.

MCF7 cells were transfected with p140Cap-RFP, fixed with 4% Paraformaldehyde, 10 min at RT, and washed twice with BSA 1% in PBS + 0.1% saponin. A blocking step with BSA 1% in PBS and 0.1% saponin was performed 1 h at RT. Cells were incubated with primary antibody anti RTN3 in BSA 1% in PBS and 0.1% saponin 1 h at RT. After two washes with BSA 1% in PBS + 0.1% saponin, cells were incubated with anti-Rabbit secondary antibody diluted in BSA 1% in PBS + 0.1% saponin 1 h at RT, washed twice with BSA 1% in PBS and 0.1% saponin, and incubated with the DNA dye DAPI (Sigma) at 0.5 μg/ml in PBS for 20 min at RT. After two more washes with PBS, a post-fixation step with PFA 4% for 2 min at RT was performed. Coverslips were mounted on slides after two washes with PBS and one wash with ddH2O.

Samples were all examined using the SP5 confocal microscope Leica equipped with four excitation laser lines (405 Diode, Argon, DPSS561, HeNe633). Images were analyzed with ImageJ software. We designed a macro that subtracts the background of both channels and applies the auto threshold Moments of Fiji. Colocalization in HeLa and MCF7 cells was performed with the plug-in JACoP (Just another Colocalization plugin) and the levels of colocalization was expressed with Manders’ coefficients M1 [45].

### Cholesterol depletion, loading, and cellular cholesterol measurement

To prepare cholesterol/methyl-β-cyclodextrin (MβCD) complexes, cholesterol was dissolved in 2-propanol/chloroform 2:1 (v/v) at a final concentration of 6.5 mM and added to an aqueous solution of 66.7 mM MβCD previously heated at 80 °C for 20 min. During the overnight evaporation of the solvent, cholesterol is progressively associated with MβCD in a soluble mixture. In cholesterol loading assays, 5 × 10^6^ cells (after overnight starvation) were incubated with 0.67 mM MβCD for 4 h, then washed; fresh medium containing 0.5 mM cholesterol/MβCD complexes was added for 24 h. In cholesterol depletion assays, cells were incubated with 0.67 mM MβCD for 4 h. After these incubation times, the cellular cholesterol was measured using the Cholesterol Fluorimetric Assay kit (Cayman Chemical, Ann Arbor, MI) as per the manufacturer’s instructions. Results were expressed as µmol cholesterol/mg cell proteins.

### Cholesterol efflux

To evaluate cholesterol efflux, 1 × 10^6^ cells (after overnight starvation) were incubated with 1 µCi/ml [^3^H] cholesterol (Amersham Bioscience) for 1 h, washed five times with PBS, and grown in a fresh medium for 24 h. The cell culture medium was collected, and cholesterol was extracted in methanol-hexane and resolved by TLC. The amount of [^3^H] cholesterol recovered was quantified by liquid scintillation and expressed as fmol/10 6 cells, according to the titration curve prepared previously. The amount of [^3^H] cholesterol in the media of mock cells was considered 100% efflux; results were expressed as percentages towards mock cells.

### ABC transporter activity

To prepare plasma-membrane vesicles enriched of ATP-binding cassette transporters, 10 × 10^6^ cells (after overnight starvation) were washed with Ringer’s solution (148.7 mM NaCl, 2.55 mM K_2_HPO_4_, 0.45 mM KH_2_PO_4_, 1.2 mM MgSO_4_; pH 7.4), lysed on crushed ice with lysis buffer (10 mM Hepes/Tris, 5 mM EDTA, 5 mM EGTA, 2 mM DTT; pH 7.4) supplemented with 2 mM PMSF, 1 mM aprotinin, 10 μg/mL pepstatin, 10 μg/mL leupeptin, and subjected to nitrogen cavitation at 1200 psi for 20 min. Samples were centrifuged at 300 g for 10 min, diluted 1:4 in the pre-centrifugation buffer (10 mM Tris/HCl, 25 mM sucrose; pH 7.5), overlaid on a sucrose cushion (10 mM Tris/HCl, 35% w/v sucrose, 1 mM EDTA; pH 7.5) and centrifuged at 14000 g for 10 min. The interface was collected, diluted 1:5 in the centrifugation buffer (10 mM Tris/HCl, 250 mM sucrose; pH 7.5), and subjected to a third centrifugation at 100000 g for 45 min (Optima L-90K Beckman Coulter Ultracentrifuge). The vesicle pellet was resuspended in 0.5 ml centrifugation buffer and stored at −80 °C until use, after the quantification of the protein content. 100 µg proteins were immunoprecipitated in non-denaturing conditions using anti-ABCA1 (diluted 1:100, mouse clone HJI, Abcam, Cambridge, UK) and anti-ABCG1 (diluted 1:100, rabbit polyclonal, #NB400-132, Novus Biologicals, Littleton, CO) antibodies, in the presence of 25 µL of PureProteome Magnetic Beads. The ATPase activity of immunopurified ABCA1 and ABCG1 was measured by a spectrophotometric method: samples (containing 20 μg proteins) were incubated for 30 min at 37 °C with 50 μl of the reaction mix (25 mM Tris/HCl, 3 mM ATP, 50 mM KCl, 2.5 mM MgSO_4_, 3 mM DTT, 0.5 mM EGTA, 2 mM ouabain, 3 mmol/L NaN_3_; pH 7.0). In each set of experiments, a blank containing 0.5 mM Na_3_VO_4_ was included. The reaction was stopped by adding 0.2 ml ice-cold stopping buffer (0.2% w/v ammonium molybdate, 1.3% v/v H2SO4, 0.9% w/v SDS, 2.3% w/v trichloroacetic acid, 1% w/v ascorbic acid). After a 30-min incubation at room temperature, the absorbance of the phosphate hydrolyzed from ATP was measured at 620 nm, using a Packard EL340 microplate reader (Bio-Tek Instruments, Winooski, MA). The absorbance was converted into μmol hydrolyzed phosphate/min/mg proteins, according to the titration curve previously prepared. ATPase activity in mock cells was considered 100%; results were expressed as a percentage towards mock cells.

### Membrane cholesterol measurement

10 × 10^6^ cells were lysed in 0.5 mL of 10 mM Tris, 100 mM NaCl, 20 mM KH_2_PO_4_, 30 mM EDTA, 1 mM EGTA, 250 mM sucrose, pH 7.5 and sonicated with 2 bursts of 10 s (Labsonic sonicator, Sartorius Stedim Biotech S.A., Aubagne Cedex, France), then centrifuged at 13000 g for 15 min at 4 °C. The supernatants were centrifuged at 100000 g for 1 h at 4 °C, using an Optima L-90K Beckman Coulter Ultracentrifuge (Beckman Coulter Inc, Fullerton, CA) to collect the membrane fractions. The pellets were resuspended in 250 µL of the assay buffer provided by fluorimetric Cholesterol/Cholesteryl Ester Assay Kit – Quantitation (Abcam) and used to measure free cholesterol in the membrane, as per manufacturer’s instructions. An aliquot of 50 µl was sonicated again to measure the membrane proteins. Results were expressed as mg cholesterol/mg membrane proteins.

### Membrane fluidity measurement

Membrane fluidity was measured in triplicate using a membrane fluidity kit (Marker Gene Technologies M0271) according to the manufacturer’s protocol. In brief, we incubated the samples (adherent or suspension cells) with 2 μM pyrenedecanoid acid (PDA) and 0.08% pluronic F127 (to increase labeling) in either Perfusion buffer or cell culture medium (supplement-free) for 30 min at 22 °C with mild shaking, then washed twice with PBS. In PBS, we recorded fluorescence emissions between 392–450 nm in 2 nm steps after excitation at 360 nm with a Tecan microplate reader (Infinite® 200 PRO NanoQuant). We subtracted a background sample where the cells were processed without PDA. With increased membrane fluidity, the lipophilic pyrene probe forms excimers upon interaction. The ratio of excimer (peak around 450 nm) to monomer (peak around 394-398 nm) IE/IM was calculated as a quantitative measure of membrane fluidity.

### Wound Healing migration assays

1×10^6^ cells were plated in 6-well plates and allowed to grow at confluence. Upon vertical linear scratch, using a 200 µl pipette tip, cells were washed twice with PBS and maintained in a serum-free medium. Images were acquired at 0 and 48 h post scratch with 10× objective Carl Zeiss microscope, and wound area closure was calculated with Fiji software. Three replicates for each condition were analyzed in at least 3 independent experiments.

### Lipid rafts flow-cytometer analysis

1 × 10^6^ cells were washed with PBS and incubated with 1 μg cholera toxin B (CTXB) Alexa Fluor-488 Conjugate (CTXB 488, Thermo Fisher Scientific) diluted in PBS for 15 min at 4 °C. At the end of incubation, cells were washed with PBS and analyzed by flow cytometry. At least 10000 events/sample were acquired by the FACSCalibur cytometer (BD Biosciences). Data analysis was performed by gating living cells and excluding death cells and debris. The mean fluorescence intensity of CTXB 488 values were calculated by subtracting the fluorescence of unlabelled cells.

### Lipid rafts isolation

Isolation of lipid rafts was performed as described in Rodgers and Rose [46]. Briefly, 100 × 10^6^ cells were lysed with 1% TritonX-100 Lysis Buffer (10 mM Tris-HC1 pH 7.5, 150 mM NaCI, 5 mM EDTA, 1 mM Na_3_VO_4_, and proteases inhibitors) and Dounce homogenized. Lysates were centrifuged for 5 min at 1300 g for nuclei and debris removal. For equilibrium centrifugation cleared lysates were diluted in an SW41 centrifuge tube with an equal volume of 85% w/v sucrose in TNEV (10 mM Tris-HC1 pH 7.5, 150 mM NaC1, 5 mM EDTA, and 1 mM Na_3_VO_4_), overlaid with 6 ml of 30% w/v and 3.5 ml 5% w/v sucrose solutions in TNEV. Samples were centrifuged for 17 h at 200000 g at 4 °C with no brake during the deceleration phase. Eleven density gradient fractions were collected and the flotillin-1 positive fractions were further assessed for Rac1 activity with the Rac1 activation assay (Cell Biolabs, Inc., San Diego, CA) according to the manufacturer’s instructions. To determine expression of the lipid-rafts marker monosialotetrahexosylganglioside (GM1), fractions were dot blotted onto a nitrocellulose membrane. The membrane was saturated with TBS 5% BSA for 2 h and incubated with HRP-conjugated cholera toxin B (1/20,000) (Sigma). Dots were revealed by chemiluminescence.

### Cell viability

Cells were seeded in 24-well plates, starved overnight, and incubated with 10 nM, 100 nM, 1 µM or 10 µM simvastatin, dissolved in dimethylsulfoxide (DMSO; final concentration: 0.1%), for 48 h. Control cells were incubated with 0.1% DMSO. Cells were stained with 5% w/v crystal violet solution in 66% v/v methanol, then washed once in deionized water. Absorbance was read at 570 nm, using a Packard EL340 microplate reader (Bio-Tek Instruments, Winooski, VT). Treatment with Doxorubicin (D1515), Vinorelbine (V2264), Paclitaxel (T7402) (Sigma Sigma-Aldrich), and combinational treatments was performed accordingly, using the indicated concentrations. The absorbance of untreated cells was considered as 100% viability; the results were expressed as a percentage of viable cells vs. untreated cells. To calculate the combination index (CI), viability was measured after incubation with simvastatin and doxorubicin as single agent in the concentration range 0.1 nM-10 mM, or with both agents at the same concentration of this range. CI, that discriminates additive, synergistic or antagonistic effects, and dose reduction index 50 (DRI50), that determines the fold of dose reduction obtained with combined drugs versus drug alone to reduce cell viability of 50%, were calculated according to [47], using the CalcuSyn software (www.biosoft.com/w/calcusyn.htm).

## Results

### p140Cap increases the metabolic flux through the MVA pathway

**Fig. 1:**
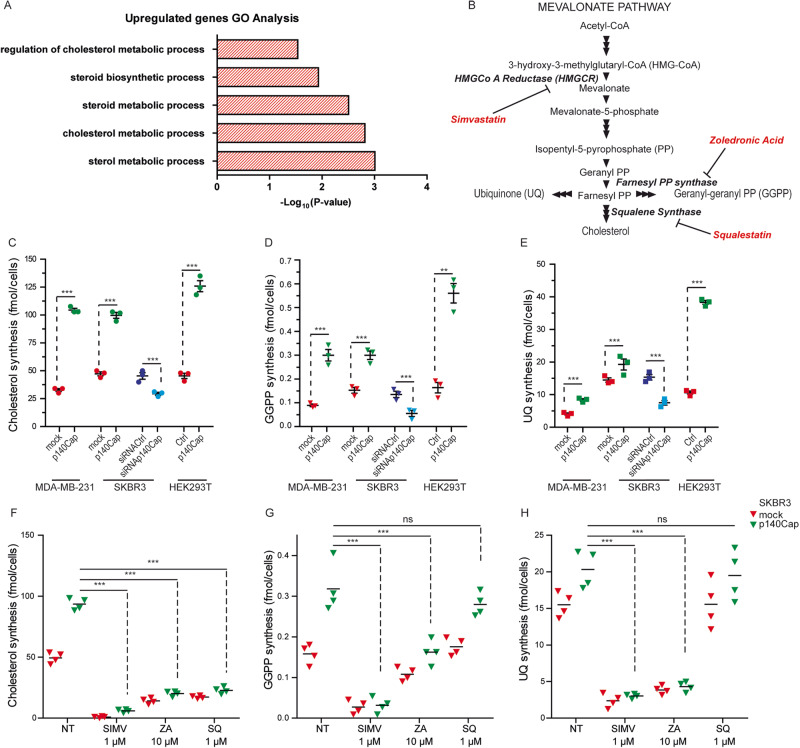
p140Cap increases the metabolic flux through the MVA pathway. **A** List of Gene Ontology terms related to cholesterol and lipid metabolism that are enriched in a dataset of up-regulated genes in MDA-MB-231 p140Cap cells. **B** Schematic representation of MVA pathway with the main intermediates, end-products, and targeted enzymes. The de novo synthesis of cholesterol (**C**), geranylgeranyl diphosphate (GGPP) (**D**), and ubiquinone (UQ) (**E**) was measured in mock and p140Cap MDA-MB-231, SKBR3, and HEK293T cells by incubating with 1 µCi [^3^H] acetate for 24 h, recovering the lipids, and measuring the radioactivity by liquid scintillation. Unpaired test (**P < 0.001; ***P < 0.0001). Error bar: SEM. **F**, **G**, **H** Cholesterol, GGPP, and UQ synthesis in SKBR3 cells treated with the indicated concentration of the MVA pathway inhibitors Simvastatin (SIMV), Zoledronic Acid (ZA), and Squalestatin 1 (SQ). ANOVA test and Bonferroni post-test (***P < 0.0001), Error bar: SEM.

To assess whether p140Cap can contribute to the metabolic landscape in BC cells, we took advantage of a gene expression dataset obtained in p140Cap-overexpressing MDA-MB-231 and control cells, previously described in [26]. We performed a Gene Ontology (GO) analysis that revealed enrichment in genes related to “sterol metabolic process” (GO:0016125) and “cholesterol metabolic process” (GO:0008203) in p140Cap cells (Fig. 1A). To investigate the role of p140Cap in the cholesterol metabolism of BC cells, we modulated p140Cap expression by gain of function (p140Cap cells) or silencing approaches, as described in Material and Methods (see Suppl. Fig. 1A for p140Cap expression), in both Her2-positive and triple-negative human BC cells, namely SKBR3 and MDA-MB-231 human cell lines [27]. Moreover, we also used two pre-clinical syngeneic mouse models, the TUBO cells, an established cell line from BALB/c-MMTV-NeuT mice, which is a model for Her2+ human BC [33], and the 4T1 cells, an highly tumorigenic and invasive mammary carcinoma model for stage IV human BC [48] (see Supplementary Fig. 1A). Therefore, we assessed the metabolic flux through the cholesterol biosynthetic pathway, also known as the MVA pathway, summarised in the schematic representation of the MVA pathway and targeted enzymes shown in Fig. 1B. We measured the synthesis of the final products cholesterol, GGPP and UQ through radiolabelling 5 × 10^6^ cells (after overnight starvation) with 1 µCi [^3^H] acetate (3600 mCi/mmol), for 24 h. We found that both MDA-MB-231 and SKBR3 p140Cap cells had a significantly increased metabolic flux through the MVA pathway compared to mock cells (Fig. 1C–E), as documented by the higher synthesis of cholesterol, GGPP and UQ. Similar results were obtained in TUBO p140Cap and 4T1 p140Cap murine cell lines (Suppl. Fig. 1B–D). Of note, when the endogenous p140Cap protein was down-regulated by RNA silencing in SKBR3 cells, the synthesis of these three metabolites was significantly lowered (Fig. 1C–E), suggesting that modulation of p140Cap levels correlates with MVA pathway activity. Interestingly, transient expression of p140Cap in HEK293T cells, a human embryonic kidney cell line (Supplementary Fig. 1A), was sufficient to increase the synthesis of cholesterol, GGPP and UQ (Fig. 1C–E), suggesting a wider spectrum of action for p140Cap, not limited to BC cells.

To assess whether the p140Cap-dependent increased synthesis of the MVA pathway metabolites was still sensitive to well-known MVA pathway drugs, we treated SKBR3 cells with the HMGCR inhibitor simvastatin (SIMV), the FPP synthase inhibitor zoledronic acid (ZA) and the squalene synthase inhibitor squalestatin (SQ) (see Fig. 1B). The synthesis of the three metabolites was reduced to similar levels between mock and p140Cap cells, according to the inhibited branch of the MVA pathway (Fig. 1F–H). These data show that the MVA pathway and the related targeted enzymes are still druggable in p140Cap cells, thus indicating that p140Cap does not constitutively activate the pathway and that p140Cap acts upstream of the HMGCR catalytic reaction.

### p140Cap controls HMGCR levels and activity through transcriptional and post-translational mechanisms

**Fig. 2:**
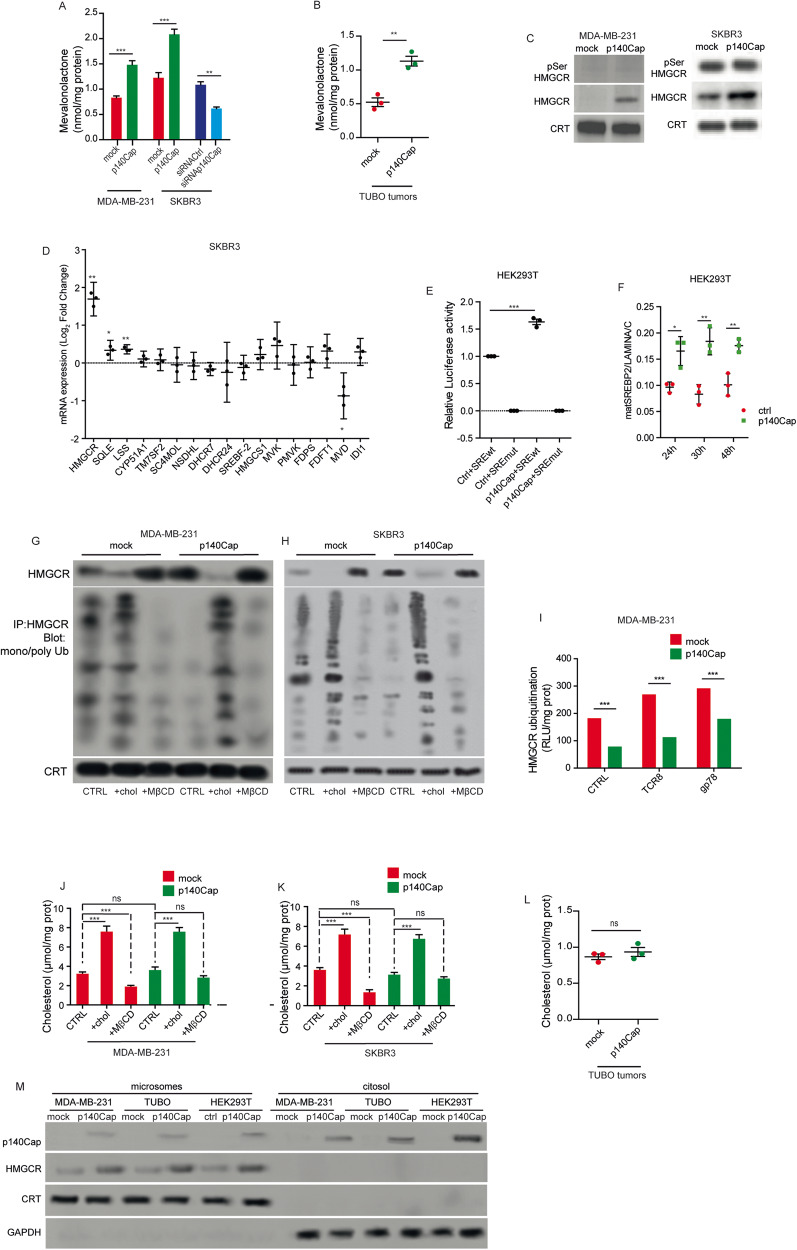
p140Cap controls HMGCR through transcriptional and post-translational mechanisms. **A** HMGCR activity in mock and p140Cap MDA-MB-231 and SKBR3 cells; 60 nCi [14 C] HMG-CoA was added to microsomal extracts. Mevalonolactone was recovered and quantified by liquid scintillation. **B** HMGCR activity in tumors derived from mock and p140Cap TUBO cells orthotopically injected in Balb/c mice. **C** Immunoblot of immunoprecipitated total HMGCR and pSer HMGCR from mock and p140Cap MDA-MB-231 and SKBR3 cells. The ER-resident protein Calreticulin (CRT) was used as a loading control of microsomal extracts. **D** Quantification of mRNA levels of genes involved in the mevalonate pathway in SKBR3 p140Cap cells relative to mock cells. **E** Analysis of SREBP2 activity by Dual Luciferase assay in HEK293T cells transiently transfected with the indicated vectors. **F** Protein levels of nuclear mature SREBP2 (mSREBP2) normalized on Lamin A/C at different time points after transfection of p140Cap in HEK293T cells (see the relative western blot in Supplementary Fig. 1I). **G**, **H** Immunoblot showing protein levels and ubiquitination of immunoprecipitated HMGCR in control cells (CTRL), following cholesterol loading (+chol) or depletion (+MβCD) experiments in MDA-MB-231 and SKBR3 cells. The ER-resident CRT protein was used as a loading control. **I** HMGCR ubiquitination levels in MDA-MB-231 through an E3 ligases activity assay. **J**, **K** Quantifications of loading depletion experiments. Cholesterol loading assays: cells were incubated with β-methyl cyclodextrin (MβCD) then fresh medium containing cholesterol/MβCD complexes was added. Cholesterol depletion assays: cells were incubated with MβCD. Cholesterol was measured using the Cholesterol Fluorimetric Assay kit and is expressed as µmol cholesterol/mg cell proteins. **L** Measurement of cholesterol levels in tumors in Balb/c mice derived from mock and p140Cap TUBO cells. **M** Immunoblot analysis of p140Cap in microsomal fractions and whole cell lysates of MDA-MB-231, TUBO and HEK293T cell lines. The ER-resident CRT protein was used as a loading control. From (**A**–**L**) Unpaired test (*P < 0.05; **P < 0.01; ***P < 0.001). Error bar: SEM.

Considering that HMGCR is the rate-limiting enzyme of the MVA pathway, we evaluated its activity by measuring the amount of [^14^C] mevalonolactone generated upon incubation with the radiolabelled isoform of the HMGCR substrate HMG-CoA. In line with the observed enhanced synthesis of the end-products of the MVA pathway, we detected a 50% increased enzymatic activity of HMGCR in MDA-MB-231 and SKBR3 p140Cap cells compared to their mock counterparts (Fig. 2A). On the other hand, p140Cap silencing in SKBR3 cells resulted in a 50% reduction of HMGCR activity (Fig. 2A). Up-regulation of HMGCR enzymatic activity was also detected in TUBO p140Cap cells and in transfected HEK293T p140Cap cells (Supplementary Fig. 1E, F). The analysis of in vivo tumors, obtained by orthotopically injection of TUBO mock or 140Cap cells in Balb-c mice, showed the up-regulation of the HMGCR in p140Cap tumors compared to mock tumors of the same volume (approximately 500 mm^3^) (Fig. 2B), indicating that the p140Cap-dependent tuning of the MVA pathway is maintained in in vivo tumor growth.

The HMGCR enzymatic activity can be negatively controlled through phosphorylation at Ser872 by the AMP-activated protein kinase (AMPK) [49]. Phosphoserine HMGCR levels were not affected in SKBR3 p140Cap cells compared to mock cells and even undetectable in MDA-MB-231 cells (Fig. 2C), suggesting that AMPK-mediated phosphorylation is not a major mechanism for regulating HMGCR in p140Cap cells. However, by WB analysis, we detected increased HMGCR protein levels in both SKBR3, MDA-MB-231 (Fig. 2C) or transiently transfected HEK293T expressing p140Cap cells (Suppl. Fig. 1G). In SKBR3 p140Cap cells, quantitative real-time PCR showed that the mRNA levels of HMGCR and to a lesser extent, of other MVA pathway genes SQLE and LSSs, were statistically significantly increased, compared to mock cells (Fig. 2D) [50].

HMGCR mRNA expression is tightly controlled at the transcriptional level according to the intracellular cholesterol content through the transcription factor SREBP2 [51]. To further investigate the activity of SREBP2 we used a reporter system in which the luciferase gene is under a SRE-containing promoter [52]. Specifically, we transiently transfected HEK293T cells with plasmids encoding p140Cap, or an empty vector, together with the luciferase reporter plasmid harbouring a wild-type (SREwt) or a mutated form (SREmut) of the SRE sequence. 24 h after transfection we detected a significant increase in luminescence signal in p140Cap versus control cells, indicating that p140Cap promotes the transcriptional activity of SREBP2 (Fig. 2E). The binding of SREBP2 to the SRE was specific since the reporter plasmid with the SREmut sequence did not induce any luminescence signal (Fig. 2E). Moreover, by WB analysis we found that the nuclear level of mature, active SREBP2 was significantly higher in p140Cap transfected HEK293T cells compared to controls (Fig. 2F and Supplementary Fig. 1H). Therefore, in p140Cap cells the increased translocation of active SRPBP2 to the nucleus likely supports its increased transcriptionally activity, explaining the luciferase assay data, and the increased levels of HMGCR mRNA and protein described above. Moreover, blocking the cleavage of SREBP2 into the Golgi with PF429242 [43], an inhibitor of the SREPB2-specific SP1 protease, the cholesterol synthesis was affected both in mock and p140Cap MDA-MB-231 cells, indicating that p140Cap acts upstream of SREBP2 cleavage at the Golgi level (Supplementary Fig. 1I).

In addition to the transcriptional regulation, HMGCR levels are also controlled by ubiquitination that promotes endoplasmic reticulum (ER)-associated proteasomal degradation to limit cholesterol production [53], as suggested by the increased ubiquitinated levels of HMGCR in cells pre-treated with the proteasome inhibitor bortezomib (Supplementary Fig. 1J). Notably, we observed that MDA-MB-231, SKBR3, and HEK293T cells expressing p140Cap consistently showed a pattern of reduced HMGCR ubiquitination compared to controls (Fig. 2G, H, Supplementary Fig. 1G), suggesting that p140Cap controls HMGCR protein stability. To further understand the mechanisms underlying the reduced HMGCR ubiquitination observed in p140Cap-expressing cells, we assessed the activity of the pool of the E3 ligases physiologically present in cell lysates and of two of them known to mediate HMGCR ubiquitination, namely Trc8 and gp78 [53]. In the presence of endogenous E3 ligases (CTRL), or of an excess of exogenous Trc8 and gp78, in MDA-MB-231 p140Cap cells HMGCR ubiquitination was lower than in mock cells (Fig. 2I), suggesting that p140Cap may control specifically E3 ligase activity involved in HMGCR ubiquitination. Overall, these data indicate that p140Cap upregulates the metabolic flux through the MVA pathway promoting the SREBP2-mediated transcription of HMGCR mRNA and maintaining high HMGCR protein levels by preventing its ubiquitination and proteasomal degradation.

To assess the ability of p140Cap cells to detect changes in cholesterol levels and degrade HMGCR accordingly, we analysed HMGCR ubiquitination status and protein levels upon cholesterol loading and depletion (Fig. 2G, H). Both cholesterol-loaded mock and p140Cap cells exhibited reduced HMGCR protein levels and increased ubiquitination, indicating that p140Cap cells retain the ability to respond to increased cholesterol levels. To deplete cholesterol at the cell plasma membrane, we treated cells with 0.67 μM methyl-β-cyclodextrin (MβCD), a well-known cholesterol chelator agent [54]. Upon MβCD treatment at this viable dose, mock cells were responsive (Fig. 2G, H), significantly decreasing cellular cholesterol and HMGCR ubiquitination and increasing HMGCR protein levels. In contrast p140Cap cells were not responsive, cholesterol levels were not lowered, and therefore HMGCR protein and ubiquitination levels remained at the same level of untreated cells (Fig. 2G, H) [55]. However, treating MDA-MB-231 cells with increasing concentrations of MβCD that do not affect significantly cell viability (from 0.67, 1, to 5 μM) (Supplementary Fig. 1K, L), we found that in p140Cap cells only the highest dose of MβCD (5 μM) was able to lower cholesterol levels, and, as a consequence, to increase the level of HMGCR (Supplementary Fig. 1M). Overall, these data indicate that p140Cap cells have a pool of membrane cholesterol not fully available for MβCD action, suggesting a different membrane composition compared to that of mock cells.

The increased cholesterol synthesis in p140Cap cells promoted either by SREBP2 activity and by HMGCR protein accumulation and increased activity, is suggestive of a condition of low intracellular cholesterol. However, p140Cap cells exhibit total cholesterol levels similar to that of mock cells (see CTRL bars in Fig. 2J, K) and similar results were also obtained in TUBO p140Cap tumors compared to mock tumors in in vivo experiments (Fig. 2L).

Finally, since the HMGCR protein is localised in the ER, to confirm p140Cap localization in the same compartment, we performed a WB analysis on MDA-MB-231, TUBO, and HEK293T p140Cap cells, which revealed that p140Cap is present in the microsomal fraction. Calreticulin (CRT) is a membrane-bound ER-resident protein that is not present in the cytosolic compartment and was used as a normalizer (Fig. 2M). To further assess the localisation of p140Cap in different cellular compartments, we analysed MCF7 and HeLa cells that allow a better confocal analysis of cytoplasmic compartments. In MCF7 cells transfected with p140Cap-GFP plasmid, confocal microscopy analysis showed a good p140Cap co-localization with two ER markers, ER tubulation factor reticulon-3 (RTN3) and Protein Disulfide Isomerases (PDI), as well as to a minor extent, with plasma membrane marker E-Cadherin. Similar experiments in HeLa cells confirm the localisation with ER marker (eSYT1-GFP Extended synaptotagmin-1), and with plasma membrane marker (PLC-Delta), while no colocalisation was found with the Golgi compartment that is brightly stained with the cis-Golgi matrix protein GM130 (Supplementary Fig. 2). Overall, both biochemical and imaging analysis show a good degree of association of p140Cap with the ER compartment, which is the major site of action of HMGCR in the regulation of the MVA pathway.

### p140Cap increases cholesterol efflux through ABC transporters

**Fig. 3:**
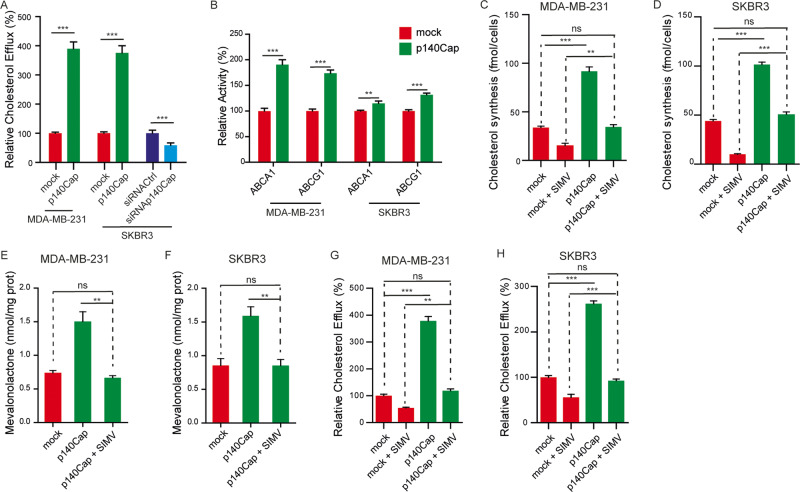
p140Cap increases cholesterol efflux through enhanced activity of ABCA1 and ABCG1. **A** Cholesterol efflux in MDA-MB-231 and SKBR3 cells. After incubation with 1 µCi/ml with [^3^H] cholesterol, cells were washed and let grow in a fresh medium for 24 h. Media was collected, cholesterol was extracted and quantified by liquid scintillation. The amount of [^3^H] cholesterol effluxed in mock cells was considered 100. **B** Evaluation of ABCA1/ABCG1 activity: ABCA1 and ABCG1 were immunoprecipitated from plasma membrane vesicles and the ATPase activity was measured by a spectrophotometric assay. The absorbance was converted into μmol hydrolyzed phosphate/min/mg proteins. ATPase activity was expressed as a percentage towards mock cells. **C**, **D** Cholesterol synthesis measurements upon 1 uM Simvastatin (SIMV) treatment for 48 h. **E**, **F** Relative HMGCR activity in cells treated with 1 uM SIMV for 48 h. **G**, **H** Cholesterol efflux measured upon 1uM SIMV treatment for 48 h. From (**A**–**H**) % ANOVA and Bonferroni post-test (**P < 0.01; ***P < 0.001). Error bar: SEM.

The data shown above indicate that the overall cholesterol content remains unchanged in p140Cap cells despite increased cholesterol synthesis by enhancing HMGCR mRNA, protein, and activity levels (Fig. 2J, K, L). Since the level of intracellular cholesterol results from the dynamic balance between synthesis, uptake, and export [5, 56], we reasoned that once synthesized, cholesterol could be rapidly effluxed from p140Cap cells to avoid the accumulation of potentially toxic excess of cholesterol. Therefore, we analysed cholesterol efflux by measuring the levels of radiolabelled cholesterol released in the medium of MDA-MB-231 and SKBR3 cells previously incubated with 1 µCi/ml [^3^H] cholesterol for 1 h, washed, and grown in a fresh medium for 24 h. p140Cap cells displayed at least a threefold increase in cholesterol efflux compared to mock cells (Fig. 3A). Similar results were obtained in TUBO, 4T1, and HEK293T cells (Supplementary Fig. 3A). Consistently, p140Cap silencing in SKBR3 cells leads to a 50% decrease in cholesterol efflux compared to control cells (Fig. 3A). In line with the increased efflux, p140Cap cells exhibited enhanced ATPase activity of the main transporters involved in cholesterol efflux, namely ATP-binding cassette A1 (ABCA1) and G1 (ABCG1) (Fig. 3B). These data suggest that although p140Cap cells synthesize more cholesterol than mock cells, the overall cholesterol content remains unchanged likely due to the enhanced ABCA1 and ABCG1-mediated cholesterol efflux.

We next asked whether the increased cholesterol synthesis observed in p140Cap cells was a consequence of the enhanced cholesterol export or vice versa. To address this question, we compared cholesterol efflux between mock and p140Cap cells with similar levels of cholesterol synthesis. To this aim, we treated p140Cap cells with 1 µM simvastatin to partially inhibit HMGCR and reduce the synthesis of cholesterol, GGPP and UQ to a level similar to mock cells (Fig. 3C, D, Supplementary Fig. 3B–E). Accordingly, upon simvastatin treatment of p140Cap cells, the HMGCR activity dropped to the level of mock untreated cells (Fig. 3E, F). In parallel with the lowered amount of synthesized cholesterol in simvastatin-treated MDA-MB-231 p140Cap and SKBR3 p140Cap cells, cholesterol efflux was reduced to a level comparable to that of untreated mock cells (Fig. 3G, H). These data suggest that the enhanced cholesterol efflux observed in p140Cap cells could result from the increased MVA pathway flux to maintain an adequate cholesterol concentration inside the cells.

### p140Cap cells show increased plasma membrane cholesterol and decreased membrane fluidity impairing BC cell migration

The ability of cancer cells to migrate and form metastasis can be affected by the plasticity of the plasma membrane [57]. Cholesterol is a crucial determinant of membrane fluidity [5], a physical property defined as the freedom of movement of a molecule within the cell membrane [5, 57].

**Fig. 4:**
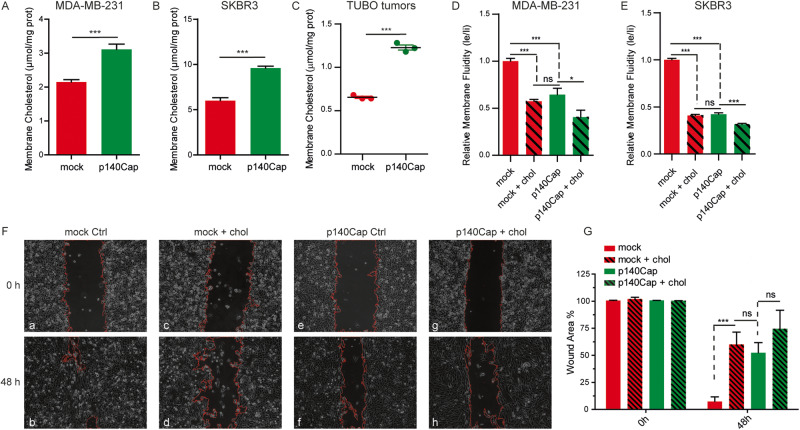
p140Cap decreases membrane fluidity impairing cell migration ability. **A**, **B** Membrane cholesterol quantification in mock and p140Cap MDA-MB-231 and SKBR3 cells. **C** Cholesterol measurement of membranes derived from TUBO mock and p140Cap tumors. **D**, **E** Membrane fluidity was measured in cholesterol loading/depletion conditions using a membrane fluidity kit according to the manufacturer’s protocol. From (**A**–**E**) ANOVA and Bonferroni post-test (*P < 0.05; ***P < 0.001; Error bar: SEM). **F** Representative images of Wound healing migration assays. 1 × 10^6^ MDA-MB-231 cells were seeded in each well and allowed to reach confluence as a monolayer. The monolayer was scratched across the center of the well and gently washed to remove the detached cells. Cells were cholesterol loaded and allowed to migrate for 48 h. **G** Quantification of wound area. ANOVA and Bonferroni post-test (*P < 0.05; ***P < 0.001; Error bar: SEM).

Given the aberrant cholesterol synthesis and the previously described impairment in migration ability observed in p140Cap cells [27], we hypothesised that p140Cap could impact cell migration by promoting cholesterol distribution to the plasma membrane and reducing membrane fluidity. To assess this hypothesis, we evaluated the amount of free cholesterol from isolated plasma membrane, and we observed a significantly increased level of plasma membrane cholesterol in human MDA-MB-231, SKBR3 (Fig. 4A, B), and murine TUBO and 4T1 (Supplementary Fig. 4A, B) cells expressing p140Cap. Similar data were obtained in membranes derived from TUBO p140Cap tumors (Fig. 4C). According to the increased plasma membrane cholesterol content, p140Cap cells also displayed decreased membrane fluidity compared to mock cells (Fig. 4D, E). As expected, by loading cholesterol into the cells, we observed a reduction in membrane fluidity in both mock and p140Cap cells. Notably, cholesterol-loaded mock cells displayed a decreased membrane fluidity similar to that of untreated p140Cap cells (Fig. 4D, E).

To further assess whether the impaired migration ability observed in p140Cap cells may depend on the reduced membrane fluidity, we performed a wound healing migration assay, modulating membrane fluidity through cholesterol loading in MDA-MB-231 mock and p140Cap cells. Notably, at 48 h upon scratch, we observed a complete wound closure by mock cells (Fig. 4F, G, panels a, b), while, as expected, p140Cap cells migrated less than mock cells, showing only a 52% closure (Fig. 4F, G, panels e, f). Cholesterol-loaded mock cells showed a percentage of wound closure similar to that observed in untreated p140Cap cells (Fig. 4F, G, panels c, d). On the other hand, cholesterol-loaded p140Cap cells migrate less than untreated cells, although the difference in wound closure was not statistically significant (Fig. 4F, G, panels g, h). Overall, these data indicate that cells expressing p140Cap display increased membrane cholesterol and reduced membrane fluidity impairing cell migration in a cholesterol-dependent manner.

### p140Cap is localized in lipid rafts and affects lipid raft-associated Rac1 signalling

**Fig. 5:**
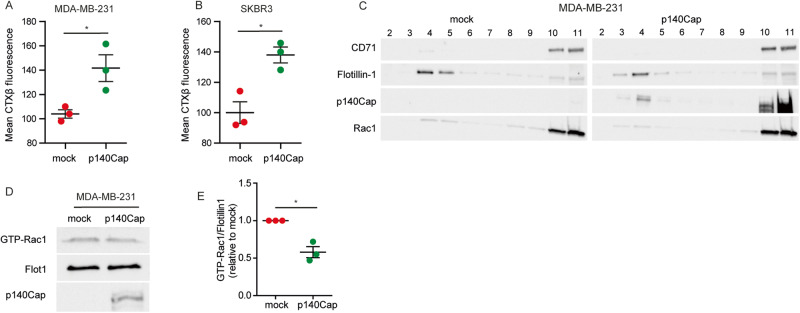
p140Cap downregulates Rac1 activity in lipid rafts. **A**, **B** Mock and p140Cap MDA-MB-231 and SKBR3 cells were labeled with Alexa CTX-β and analyzed by flow cytometry. Quantification was performed considering fluorescence mean intensity for each sample. **C** Immunoblot of fractions recovered from a sucrose gradient ultracentrifuge of MDA-MB-231 cells. Triton-X 100 insoluble fractions were identified by flotillin-1, whereas TritonX-100 insoluble fractions are positive for CD71. **D** Rac1 Activation Assay performed on the lipid-raft enriched fraction (#4) of MDA-MB-231 cells. Representative immunoblot of the pull-down experiment with Rac1, WB of flotillin-1, and p140Cap. **E** Densitometric analysis of relative GTP-Rac1 levels, normalized on flotillin-1. In (**A**, **B**, **E**) ANOVA and Bonferroni post-test (*P < 0.05; ***P < 0.001; Error bar: SEM).

Cholesterol plays a crucial role in the organization of lipid rafts, which are small (10-200 nm), highly dynamic, sterol- and sphingolipid-enriched domains at the plasma membrane that act as organizing centres for the assembly of signalling molecules, regulating cellular signalling and biological processes, including cell migration [58–60]. We investigated whether p140Cap could affect the organisation of lipid rafts by analysing the fluorescent signal of cholera toxin B (CTXB), a well-known lipid raft marker that recognizes and binds the glycolipid receptor ganglioside molecule GM1 [61]. Flow-cytometry analysis on CTXB-labelled cells revealed an increase in the mean intensity of CTXB fluorescence in MDA-MB-231 and SKBR3 p140Cap cells compared to mock cells, indicating an increase in the lipid raft marker in p140Cap cells (Fig. 5A, B).

We have previously shown that p140Cap exhibits scaffolding functions at the plasma membrane and modulates protein localization at lipid rafts in neurons [62, 63]. Therefore, we evaluated p140Cap lipid raft localization in MDA-MB-231 p140Cap cells via WB analysis of isolated TritonX-100 insoluble membrane fractions obtained through a sucrose density gradient centrifugation [46]. Fractions enriched in flotillin-1 (fractions 4-5) contain lipid rafts, whereas transferrin receptor (CD71)-positive fractions (fractions 10-11) represent the non-lipid rafts compartment (Fig. 5C). To further confirm the quality of our lipid rafts isolation we also assessed through dot blot assay the presence of CTBX, which was specifically found in fractions 4-5 (See Suppl. Fig. 4C). The results revealed that p140Cap is present in non-lipid raft fractions, as expected, but also in flotillin- and CTBX-positive fractions (fraction 4), indicating that in BC cells p140Cap is a component of the lipid rafts. These data suggest its putative ability to modulate signalling events related to lipid rafts domains. We have already shown that p140Cap curbs Rac1 activity by negatively regulating its GEF Tiam1 in Her2-amplified BC cell lines [27, 62]. Accordingly, we assessed whether p140Cap could modulate Rac1 activity in MDA-MB-231 lipid raft compartment [64]. Immunoblot analysis of the active Rac1 pull-down revealed that the level of active Rac1 relative to flotillin-1 is lower in lipid rafts derived from p140Cap than mock MDA-MB-231 cells (Fig. 5D, E). Overall, p140Cap is also a component of the lipid rafts compartment in BC cells, where it could participate in the negative control of Rac1 activity, whose inhibition may contribute to the observed impaired cell migration in p140Cap BC cells.

### p140Cap increases cell sensitivity to statins treatment

There is emerging interest in exploiting statins in anticancer therapy, since there is preclinical evidence of their antiproliferative, proapoptotic, and anti-invasive properties [65–67]. Moreover, clinical data on BC patient cohorts show that statins can reduce the percentage of patients who experience relapse after therapy and thereby increase BC survival [18, 19].

**Fig. 6:**
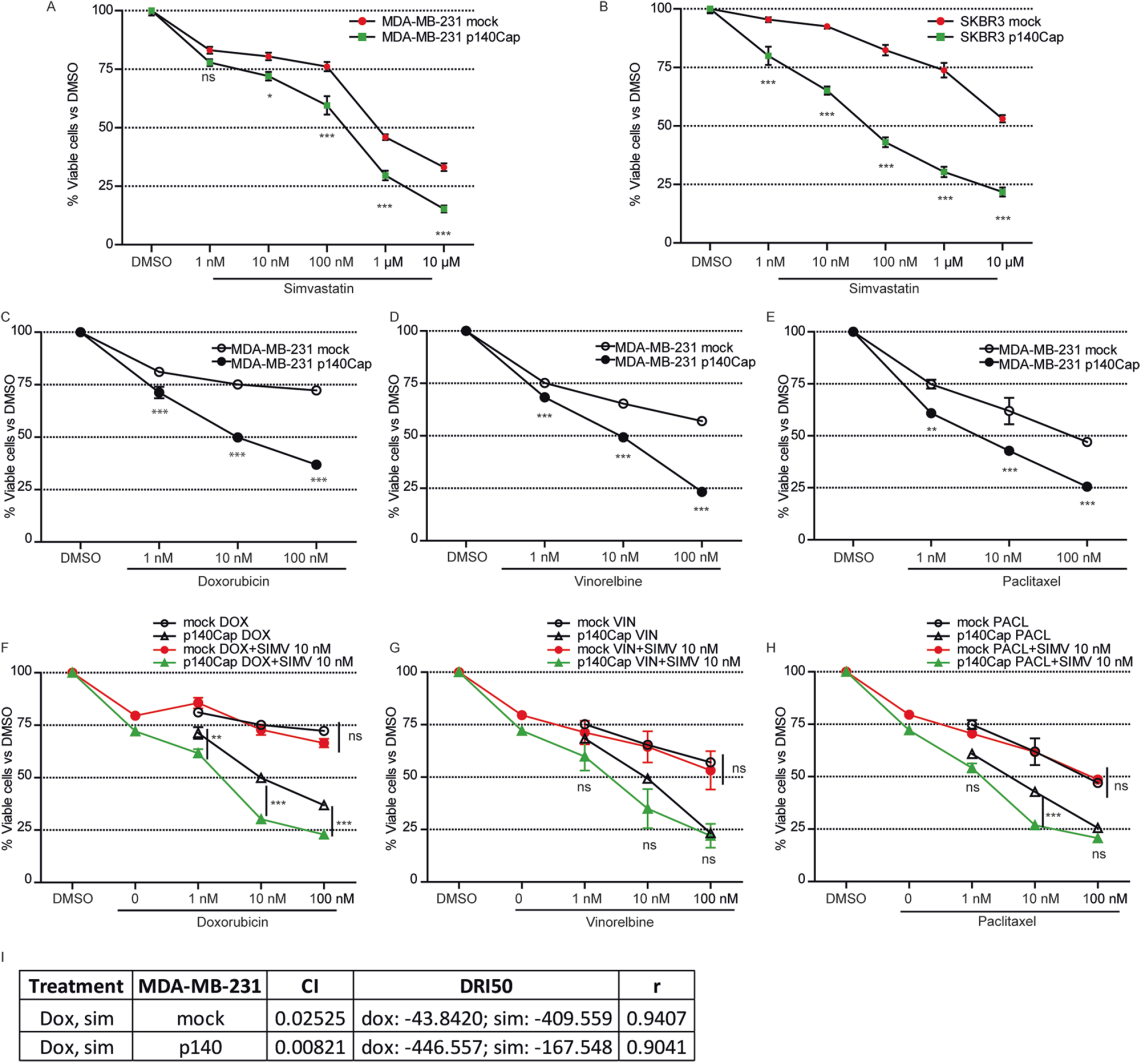
p140Cap increases cell sensitivity to statins treatment. **A**, **B** Fraction of viable cells upon simvastatin treatment. The absorbance of untreated cells was considered 100%; the results were expressed as a percentage of viable cells treated with SIMV vs. untreated cells. **C**, **D**, **E** Viability assay quantification of cells treated with chemotherapeutic agents or the combinational treatment (**F**, **G**, **H**) with chemotherapeutic agents and SIMV at the indicated concentrations. ANOVA (*P < 0.05; **P < 0.01; ***P < 0.001). Error bar: SEM. **I** Combination index upon combined treatment of Doxorubicin and Simvastatin at indicated concentrations: CI < 1 indicates a synergistic effect. DRI50 is the dose reduction index. CI was calculated with the CalcuSyn software (www.biosoft.com/w/calcusyn.htm).

Hence, we asked whether p140Cap-mediated modulation of the MVA pathway could affect cell viability upon statin treatment. To this end, we treated MDA-MB-231 and SKBR3 mock and p140Cap cells with increasing concentrations of simvastatin and measured the cell viability after 48 h. Statin treatment reduced cell viability in both mock and p140Cap cells, but MDA-MB-231 and SKBR3 p140Cap cells were more sensitive to statins than mock cells in a range of concentrations from 1 nM to 10 μM of simvastatin (Fig. 6A, B and Supplementary Fig. 5A). Similar results were obtained in MDA-MB-231 and TUBO cells treated with Atorvastatin, a commonly used cholesterol-lowering drug [68, 69] (Supplementary Fig. 5B, C).

In vitro studies have shown that statins can decrease the cell viability of various cancer cell lines through different molecular mechanisms, including cholesterol deprivation [70]. To test whether cholesterol reduction contributes to the increased susceptibility to simvastatin of p140Cap cells, we performed rescue experiments on cell viability by loading statin-treated p140Cap cells with cholesterol. Indeed, cholesterol loading in simvastatin-treated p140Cap cells partially rescued the cell viability in both human and murine cell lines, suggesting an enhanced metabolic dependency linked to the availability of cellular cholesterol (Supplementary Fig. 5D, E). Therefore, the increased sensitivity of p140Cap cells to the inhibition of the MVA pathway is likely dependent on their augmented metabolic dependency to cholesterol.

Statins appear to increase the efficacy of conventional anticancer treatments in patients [5, 71, 72]. From the above results, we expected that p140Cap cells could display increased sensitivity compared to mock cells, to a combination of chemotherapeutic drugs and statins. To assess this hypothesis, first, we analysed the effect of three selected chemotherapeutic drugs commonly used in BC treatment - doxorubicin, vinorelbine, and paclitaxel - on cell viability and we found that MDA-MB-231 p140Cap cells were more sensitive to the treatments than mock cells, with a significantly decreased viability at 1 nM concentrations (Fig. 6C–E). Then, we treated BC cells with low simvastatin concentration (10 nM) in combination with each one of the three selected drugs. The combined treatment of 10 nM simvastatin and increasing concentrations of doxorubicin (1 to 100 nM), or paclitaxel (10-100 nM) further significantly decreased cell viability of p140Cap cells, compared to chemotherapeutics alone. In contrast, the combined treatment in mock cells was not effective (Fig. 6F–H). To clarify whether this effect was due to an additive or a synergistic effect, we performed dose-response experiments, incubating mock and p140Cap MDA-MB-231 cells with increasing concentrations of doxorubicin (from 0.1 nM to 10 mM), alone or in combination with the same concentration range of simvastatin. The combined treatment was synergistic, because the Combination Index (CI) values computed for the different combinations of drugs were <1 in all the experimental settings (Fig. 6I). The effect was more pronounced in p140Cap than mock cells. Dose reduction index 50 (DRI50) quantifies the extent of dose reduction obtained for the 50% viability inhibitory effect in combination setting in comparison to each drug alone. The DRI50 values suggested that the combination of simvastatin plus doxorubicin sensitizes cells to doxorubicin, considered as single agents, 10-fold more in p140Cap cells than in mock cells (Fig. 6I). Overall, these data show that p140Cap BC cells show enhanced sensitivity to statin and chemotherapeutic treatments either alone or in combination at low concentrations, suggesting that adjuvant treatment with statins could be particularly advantageous in BC patients with p140Cap-expressing tumors.

## Discussion

**Fig. 7:**
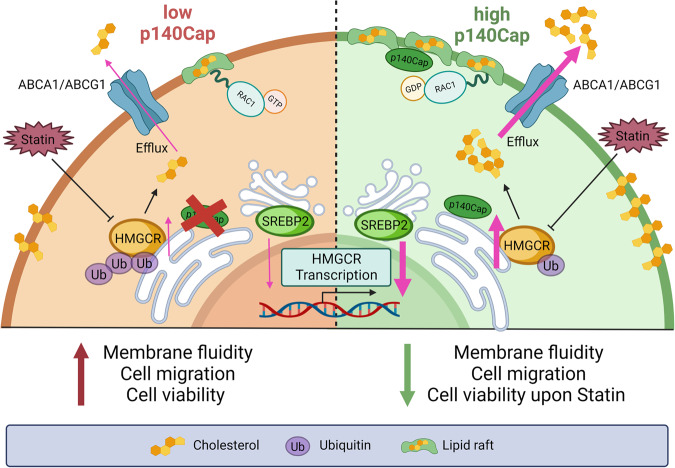
Schematic representation of p140Cap effects in cholesterol metabolism of BC cells. p140Cap expressing cells exhibit upregulated activity of HMGCR due to increased SREBP2 transcription factor activity and HMGCR protein stability. p140Cap associates to the ER compartment. Moreover, p140Cap cells show enhanced cholesterol efflux and increased cholesterol localization to the plasma membrane, leading to reduced membrane fluidity and cell migration ability. p140Cap also associates with lipid rafts, negatively regulating Rac1 activity. Finally, p140Cap expression decreases cell viability upon statin, conventional chemotherapeutic agents, and combined treatment.

In this work, we identify the p140Cap adaptor protein as a new player in the control of the MVA pathway and cholesterol metabolism in BC cells. In particular, we demonstrated that cells and tumors that express p140Cap show increased synthesis of the MVA products by the upregulation of the rate-limiting enzyme of the MVA pathway, HMGCR, therefore affecting tumor cell properties such as cell migration and sensitivity to drug treatments, upon combinatory treatments with low dose statins and conventional chemotherapeutic drugs (Fig. 7).

Cholesterol synthesis has been extensively described as upregulated in several malignancies, including BC [4, 73], since it is mostly used as a building block for proliferating cells [5]. Thus, our data showing the ability of p140Cap to enhance cholesterol synthesis appear to point to a pro-tumorigenic effect, in contrast with the previously described role of p140Cap as a tumor suppressor protein that counteracts tumor progression [27, 29]. However, cholesterol balance and intracellular localisation may influence several aspects of the plasma membrane biology, thus controlling various cancer cell features [74]. For instance, reduced plasma membrane cholesterol can increase membrane fluidity, leading to enhanced cell migration, which might eventually promote metastasis dissemination [57, 75]. Our data show that the increased newly synthesized cholesterol in p140Cap cells does not accumulate inside the cells, but it is either rapidly extruded by ABC transporters or distributed to the cell membrane, leading to reduced membrane fluidity [57]. Both a high p140Cap status [27] and a low membrane fluidity [57, 76, 77] have been previously demonstrated to be associated with a reduced BC cell migration. Our data demonstrate that cholesterol enrichment in the cell membrane in p140Cap cells might contribute to the increased membrane rigidity and the decreased migration ability of p140Cap cells. Indeed, by loading mock cells that express low or undetectable levels of p14Cap with cholesterol we were able to decrease membrane fluidity to the same extent as p140Cap cells, leading to reduced cell migration. Moreover, we also show that p140Cap can localize in the lipid raft compartment of BC cells, negatively regulating lipid raft-associated Rac1 activation. Overall, these data highlight an unexpected role of p140Cap in cholesterol metabolism in BC, involved in regulation of cell migration.

p140Cap exerts this effect on the MVA pathway both in Her2-amplified (SKBR3, TUBO) and in triple-negative (MDA-MB-231, 4T1) BC cellular models, suggesting a general role of p140Cap in different molecular BC subtypes. When HMGCR was inhibited by simvastatin, the production of cholesterol, GGPP, and ubiquinone was significantly reduced in p140Cap cells, suggesting that p140Cap acts upstream of the HMGCR activity. Notably, the effects observed in vitro, were also consistently reproduced in in vivo conditions, showing an increased HMGCR activity in p140Cap tumors grown in syngenic Balb-c mice.

Among the several mechanisms that can regulate HMGCR levels and activity, our data show that p140Cap finely tunes HMGCR at both transcriptional ad post-transcriptional levels. Indeed, we observed an increase level of HMGCR mRNA, likely through the increased transcriptional control exerted by the SREBP2 transcription factor, and an increased level of HMGCR protein. In physiological conditions, cells modulate SREBP2 maturation and HMGCR ubiquitination according to the intracellular cholesterol levels sensed in the ER [3, 4]. Specifically, low cholesterol levels in the ER initiate a feedback mechanism leading to both SREBP2 maturation and nuclear translocation and reduced ubiquitin-dependent HMGCR degradation, thus increasing cholesterol biosynthesis. Notably, the active form of SREBP2 is more translocated into the nucleus in p140Cap cells than in mock cells. Moreover, in p140Cap cells, HMGCR is less ubiquitinated at the level of ER, leading to increased protein stability. Indeed, p140Cap was detected in the microsomal fraction containing ER membranes, the specific site of action of HMGCR. HMGCR ubiquitination is reduced in p140Cap cells also in the presence of specific ER-dependent E3 ligases such as Trc8 and gp78, reinforcing the idea that p140Cap acts at the level of the ER by affecting E3 ligase activity. Although further studies are required to better understand how the presence of p140Cap at the level of the ER might modulate E3 ligases and the HMGCR activity, these data indicate that p140Cap cells might sense low cholesterol levels in the ER, hence promoting the dual control mechanisms of HMGCR.

It has been already well described that mutant p53 associates with SREBP2 and upregulates HMGCR and other MVA genes in BC cells, making the cells highly responsive on the flux through the MVA pathway and sensitive to its inhibition [78]. The main cell lines used in our work harbour different missense mutations of p53 (p.R280K for MDA-MB-231 and p.R175H for SKBR3) (https://www.atcc.org/, https://cancer.sanger.ac.uk/cell_lines). However, we observed that the increased metabolic flux through the MVA pathway by p140Cap is also present in HEK293T and 4T1 cell lines that harbour a wt-p53 and a truncated, non-functional form of p53, respectively. Thus, our data strongly suggest that p140Cap exerts a mechanism of control of the MVA pathway independently on mutant p53 activity.

Cholesterol synthesis and efflux are strictly interconnected processes involved in maintaining cellular homeostasis [56]. Indeed, we found that reducing cholesterol synthesis and HMGCR activity by statin treatment in p140Cap cells determines a parallel decreased efflux, indicating that the increased efflux is a consequence of the observed MVA pathway up-regulation and not vice versa. Our data also suggest that the enhanced outflow from the cells is due to a homeostatic mechanism to preserve an adequate intracellular concentration of cholesterol. In particular, we showed that both ABCA1 and ABCG1 transporters were more active in p140Cap cells.

Cell membrane fluidity describes the freedom of movement of the cell membrane constituents. Several studies underline how both biophysical and biochemical factors may affect cell fluidity. Among these, cholesterol is a key molecule able to increase membrane rigidity [79, 80]. p140Cap expression is accompanied by an increased cholesterol content in the membrane and the subsequent reduction of membrane fluidity. The role of cholesterol metabolism in BC development is still controversial since studies on patient cohorts failed to provide a consensus regarding the association between cholesterol and BC [4, 5, 73]. However, previous studies show that reduced cholesterol in the membrane of triple-negative breast cancer (TNBC) cells is associated with increased metastatic potential [81]. In a subgroup analysis of the patients of the TGCA cohort, stratified for the different BC molecular subtypes (i.e. luminal, Her2+ and triple-negative-TNBC), we recently reported that, compared to luminal and Her2+ patients, TNBC patients showed significantly reduced expression levels of *SRCIN1* transcript and p140Cap protein levels, indicating that BC with clinico-pathological features of aggressiveness, such as TNBCs, display comparatively lower level of the tumor suppressor p140Cap [31]. The present data indicating that p140Cap is a new player in the regulation of plasma membrane fluidity, may add a further layer of complexity to the stratification of TNBC, where the levels of p140Cap may be used as clinical biomarker for tumor aggressiveness.

Here we also found that p140Cap localizes in the lipid raft compartment of BC cells, negatively regulating Rac1 activation. These data are in line with our previous findings in the HER2-amplified cellular model [62]. Moreover, p140Cap localisation was also found in synaptic lipid rafts in neurons, controlling the recruitment of GluN2A and PSD95, reinforcing the hypothesis that p140Cap has a role in lipid raft protein organization and functionality [63].

In our experimental setting, we also demonstrate that the presence of p140Cap can amplify the sensitivity of BC cells to statin treatment. We demonstrate that statin inhibition of upregulated HMGCR in p140Cap expressing cells leads to decreased cell viability. The therapeutic potential of statins on survival and proliferation of cancer cells has been extensively reported in the literature [5, 82], also as adjuvant therapy with a chemotherapeutic agent [69, 83]. For instance, it has been demonstrated that Atorvastatin was effective in enhancing the chemotherapeutic effect of doxorubicin in TNBC and in leukemia [69, 83]. Here, we show that in BC p140Cap plays a crucial role in reducing cell viability upon statins or conventional chemotherapeutic drugs, including doxorubicin, paclitaxel, and vinorelbine. Finally, we also show that cells expressing p140Cap are more sensitive to combined treatment with statin and chemotherapeutic drugs at low doses. Dose-response experiments with simvastatin and doxorubicin combination showed synergistic effects in the Combination Index (CI) values, with a greater effect in p140Cap than mock cells. The DRI50 values suggest that the combination of simvastatin plus doxorubicin sensitizes cells to doxorubicin, considered as single agents, 10-fold more in p140Cap cells than in mock cells. This is consistent with our previous data, showing that in neuroblastoma models, p140Cap cells show significantly increased sensitivity to low doses of doxorubicin and etoposide, two common drugs employed in neuroblastoma treatment [29]. Taken together we highlight the essential role of p140Cap in BC cells, in terms of drug responsiveness.

Overall, our data highlight a new unexpected role for p140Cap in BC cells, namely its ability to regulate the MVA pathway, therefore influencing tumor cell properties such as cell migration and sensitivity to drug treatments (Fig. 7). In line with previous data showing the tumor-suppressor function of p140Cap, these results also highlight p140Cap as a key regulator of cell viability to conventional therapeutics and combined treatment with statins, thus paving the way to the use of p140Cap as a potent biomarker to stratify patients for possible new therapeutic options.

### Reporting summary

Further information on research design is available in the Nature Research Reporting Summary linked to this article.

### Supplementary information

Supplementary Figure 1

Supplementary Figure 2

Supplementary Figure 3

Supplementary Figure 4

Supplementary Figure 5

Supplementary figures legends

Reporting Summary

Western Blot uncropped images

rt_PCR

GO Analysis

## Data Availability

The main data generated or analysed during this study are included in this published article (and its Supplementary Information files). The datasets generated during and/or analysed during the current study are available from the corresponding author on reasonable request.
