# Supplementary figures and images for: p140Cap modulates the mevalonate pathway decreasing cell migration and enhancing drug sensitivity in breast cancer cells
Source: Cell Death Dis. 2023 Dec 20;14(12):849. doi: 10.1038/s41419-023-06357-z (PMC10733353; doi:10.1038/s41419-023-06357-z)

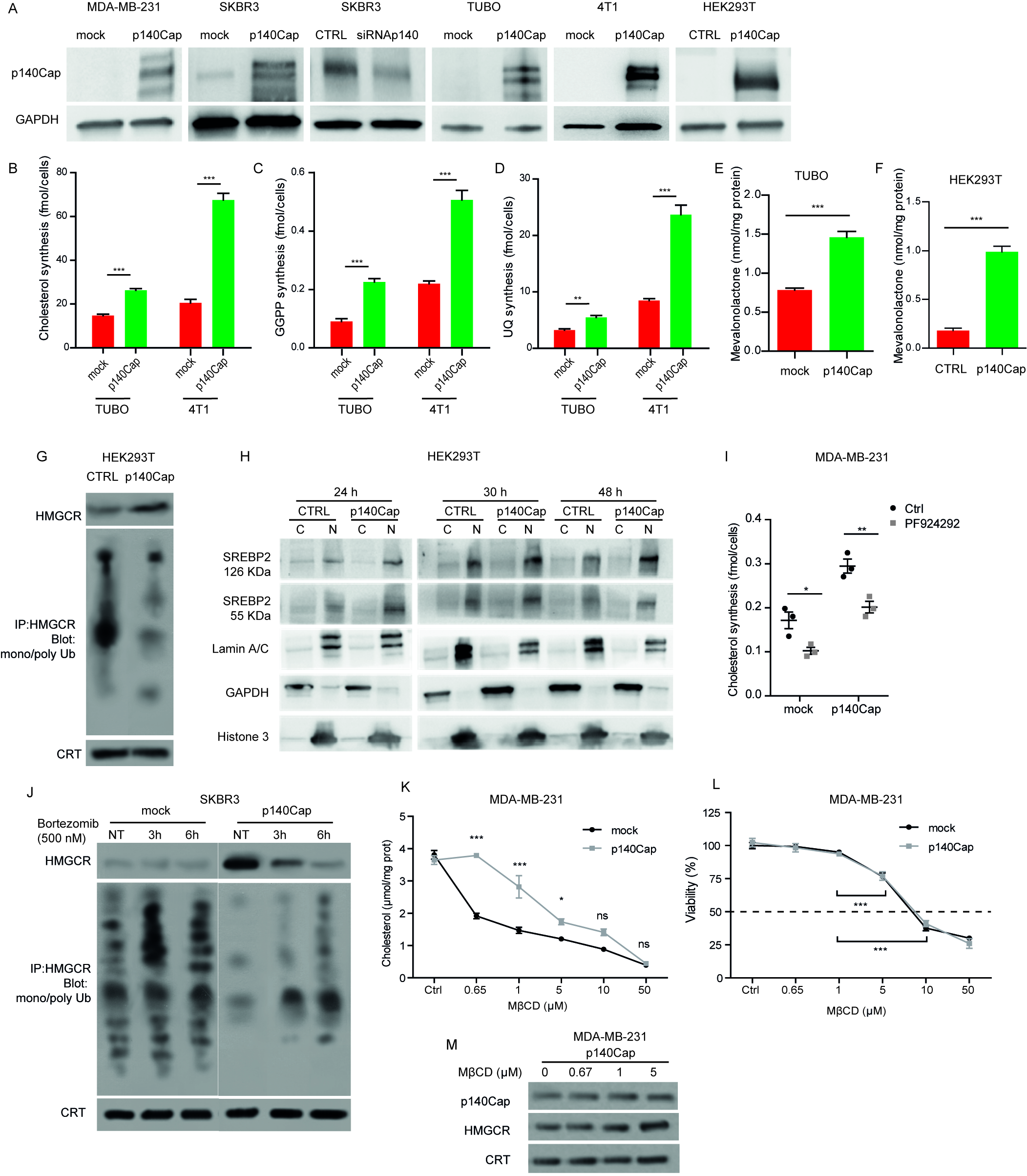

Supplement: Supplementary file 1 — Supplementary Figure 1 [file 41419_2023_6357_MOESM1_ESM.tif]

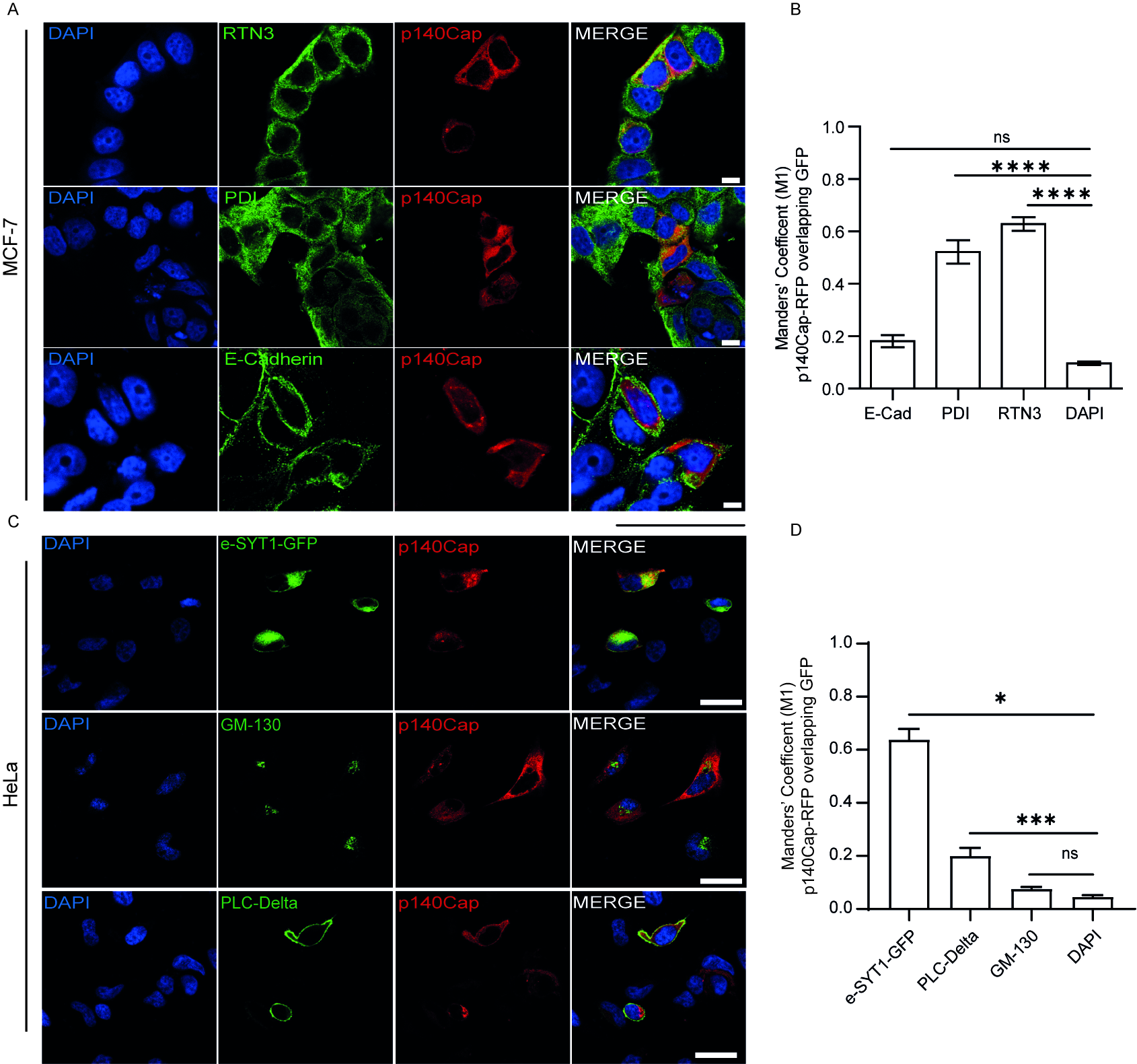

Supplement: Supplementary file 2 — Supplementary Figure 2 [file 41419_2023_6357_MOESM2_ESM.tif]

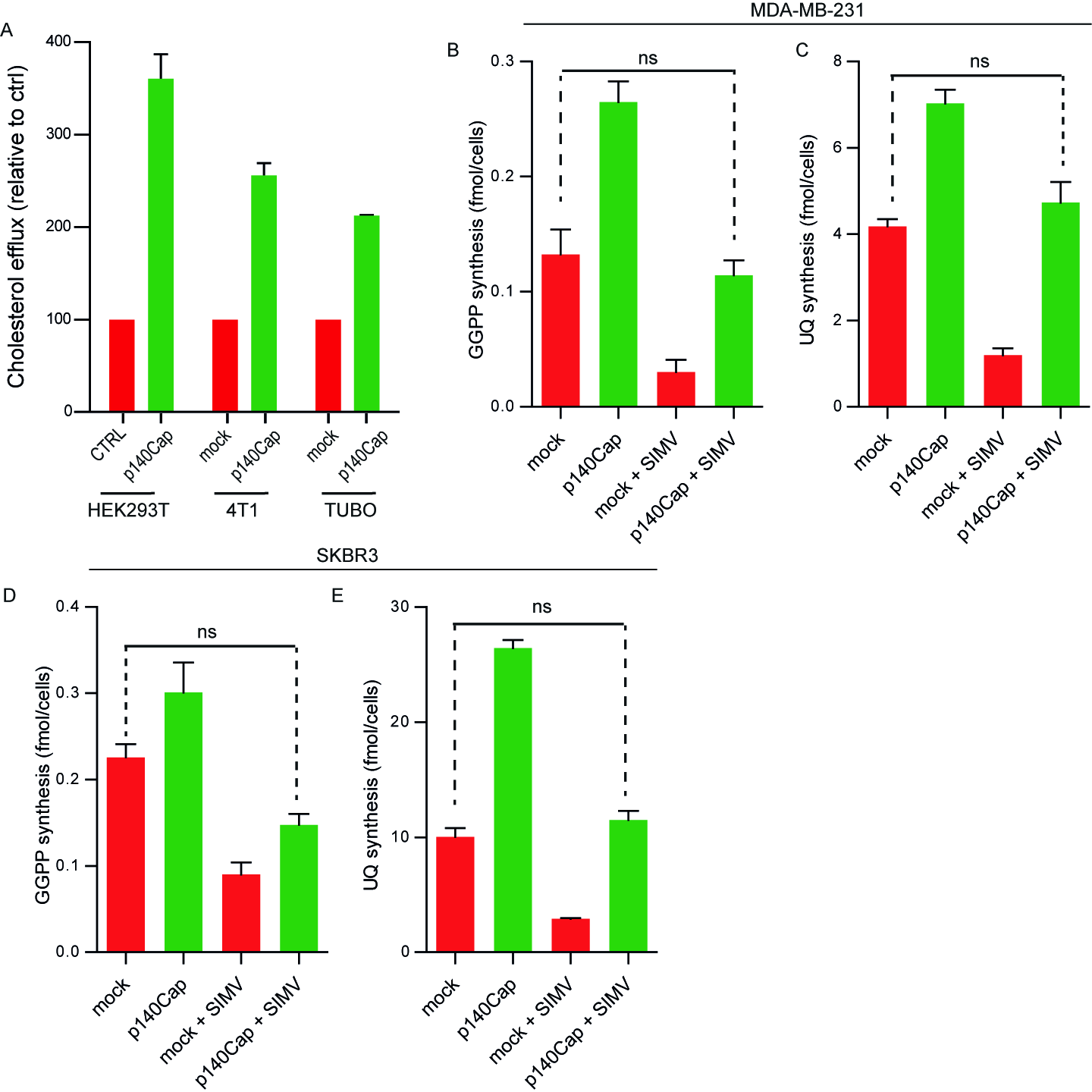

Supplement: Supplementary file 3 — Supplementary Figure 3 [file 41419_2023_6357_MOESM3_ESM.tif]

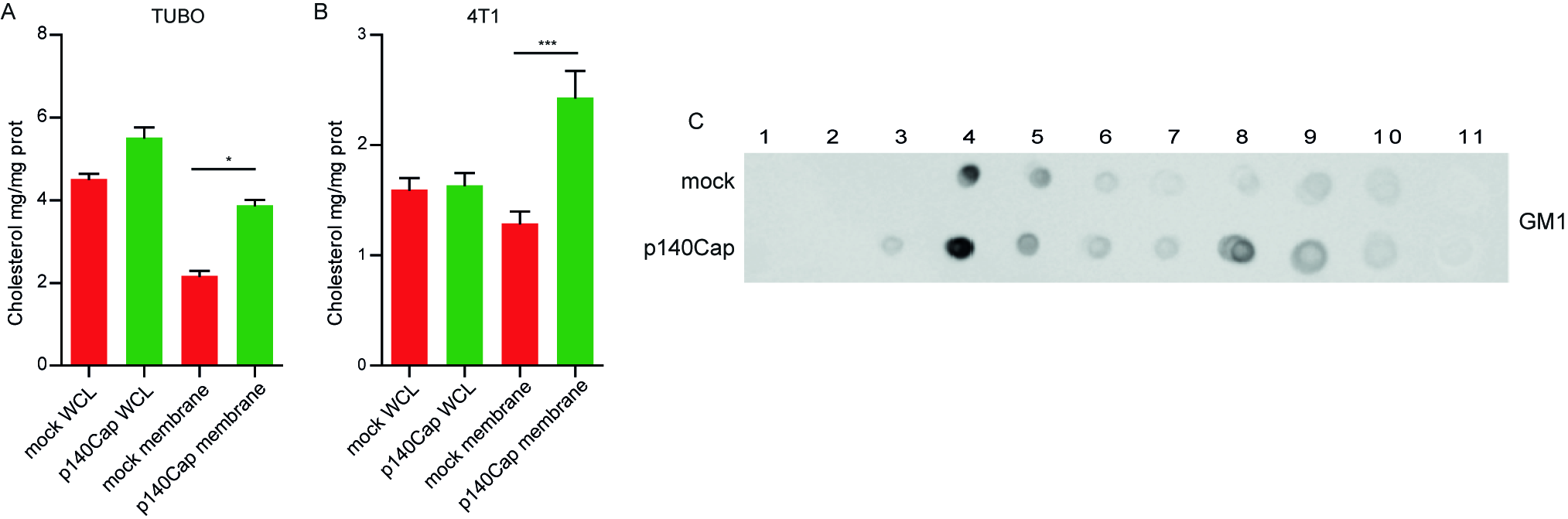

Supplement: Supplementary file 4 — Supplementary Figure 4 [file 41419_2023_6357_MOESM4_ESM.tif]

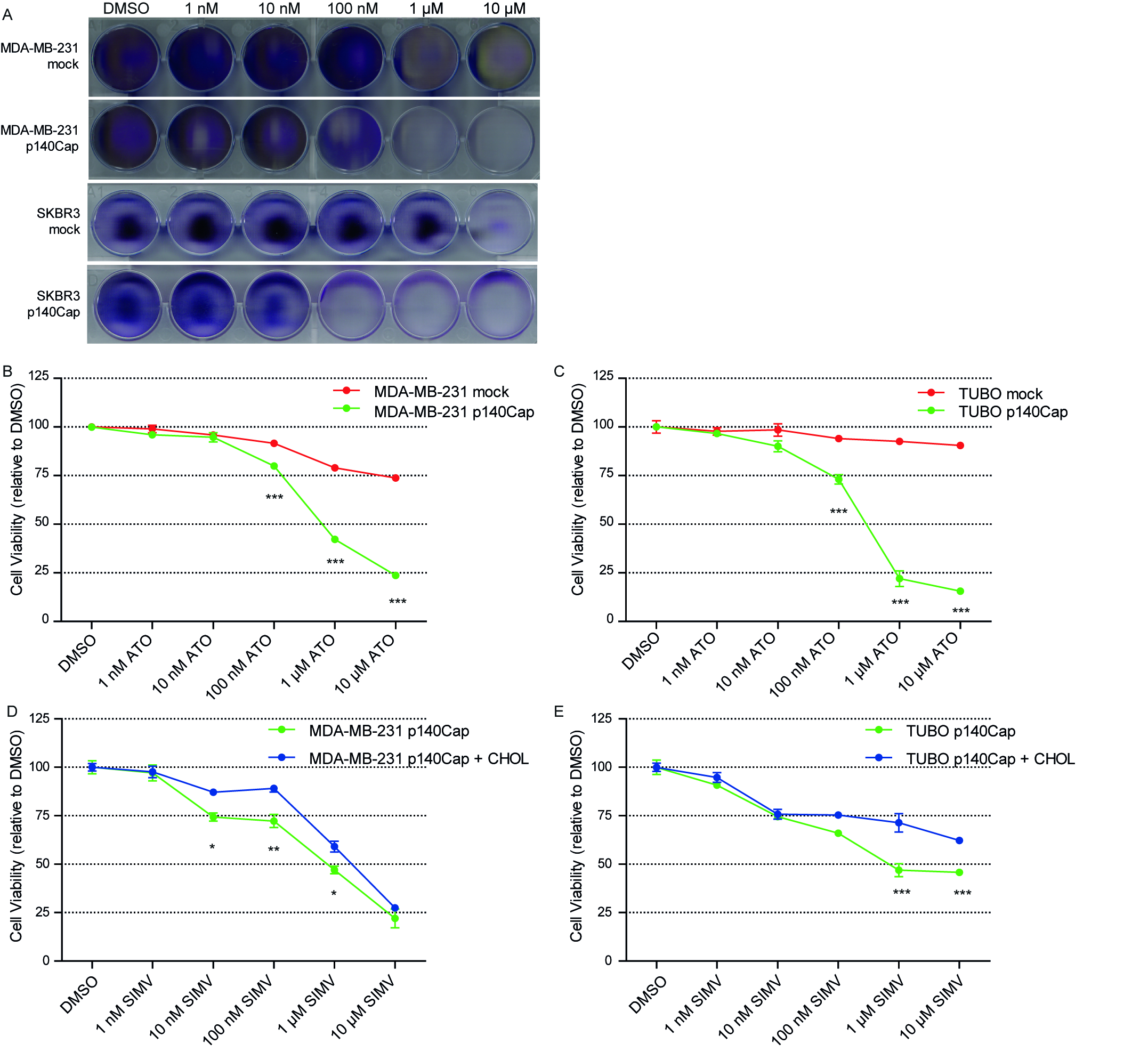

Supplement: Supplementary file 5 — Supplementary Figure 5 [file 41419_2023_6357_MOESM5_ESM.tif]
