# Supplementary material for: p140Cap modulates the mevalonate pathway decreasing cell migration and enhancing drug sensitivity in breast cancer cells
Source: Cell Death Dis. 2023 Dec 20;14(12):849. doi: 10.1038/s41419-023-06357-z (PMC10733353; doi:10.1038/s41419-023-06357-z)
Supplement: Supplementary file 6 — Supplementary figures legends [file 41419_2023_6357_MOESM6_ESM.docx]

**Supplementary Figure 1**

**(A)** Immunoblot of p140Cap in MDA-MB-231, SKBR3, TUBO, 4T1, and HEK293T cell lines. GAPDH was used as a loading control. **(B-D)** Analysis of the de novo synthesis of cholesterol, GGPP, and UQ in the murine BC cell lines TUBO and 4T1 cells. Cells were radiolabeled with 1 µCi [^3^H]acetate for 24 h. The radioactivity of the lipid molecules was measured by liquid scintillation. Cholesterol, GGPP, and UQ synthesis are expressed as fmoles/10^6^ cells. Unpaired test (**P<0.001; ***P<0.0001). Error bar: SEM. **(E)** HMGCR activity in TUBO and **(F)** HEK293T cells; 60 nCi [^14^C] HMG-CoA was added to microsomal extracts, and the labeled product Mevalonolactone was recovered and quantified by liquid scintillation. Unpaired test (***P<0.001). Error bar: SEM. **(G)** Immunoblot showing protein levels and ubiquitination of immunoprecipitated HMGCR upon transfection in HEK293T cells with the p140Cap construct or empty vector. Calreticulin (CRT) was used as loading control of microsomal extracts. **(H)** Immunoblot of SREBP2 total (126 kDa) and mature (55 kDa) proteins in nuclei (N) and cytosol (C) of HEK293T cells at 24, 30, and 48 hours upon transfection cells with the p140Cap construct or empty vector. **(I)** Cholesterol synthesis in MDA-MB-231 upon treatment with SP1 inhibitor PF924292. **(J)** Immunoblot showing protein levels and ubiquitination of immunoprecipitated HMGCR following proteasomal inhibition with 500 nM Bortezomib for the indicated time in SKBR3 cells. **(K)** Cholesterol levels in mock and p140Cap MDA-MB-231 cells upon treatments with the indicated concentrations of MβCD for 4 h. Cholesterol level was measured by a Fluorimetric Assay kit (Cayman Chemical, Ann Arbor, MI) according to manufacturer’s instructions. Two-Way ANOVA (*P<0.005; ***P<0.0001). **(L)** Percentage of viable cells upon MβCD treatment shown in K. **(M)** Immunoblot of p140Cap, HMGCR, and Calreticulin (CRT) in the microsomal fraction of MDA-MB-231 cells treated with the indicated concentration of MβCD.

**Supplementary Figure 2**

**(A, B)** Confocal images and co-localization analysis of p140Cap and markers of the ER (RTN3 and PDI), plasma membrane (E-Cadherin), and nucleus (DAPI) in MCF-7 cells. Scale bars 10 µm. **(C, D)** Confocal images and co-localization analysis of p140Cap and markers of the ER (e-SYT1-GFP), the Golgi apparatus (GM-130), and the PM (PLC-Delta) in HeLa cells. Scale bars 10 µm.

**Supplementary Figure 3**

**(A)** Cholesterol efflux in HEK293T, 4T1, and TUBO cells. After incubation with 1 µCi/ml with [^3^H]cholesterol, cells were washed and let grow in a fresh medium for 24 h. Media was collected, and cholesterol was extracted and quantified by liquid scintillation. **(B-E)** GGPP and UQ synthesis in MDA-MB-231 and SKBR3 cells treated with 1 uM simvastatin (SIMV) for 48h. Statistical test: ANOVA.

**Supplementary Figure 4**

**(A, B)** Cholesterol measurement in the whole cell lysate (WCL) and in membranes of TUBO and 4T1 cell lines. **(C)** Dot bot of the fractions obtained by sucrose density gradient ultracentrifugation of mock and p140Cap MDA-MB-231 lysates. Fractions were dot blotted with Cholera toxin B (CTXB), a well-known lipid raft marker that recognizes and binds the glycolipid receptor ganglioside molecule GM1.

**Supplementary Figure 5**

**(A)** Representative images of crystal violet viability assay of mock and p140Cap MDA-MB-231 or SKBR3 cells incubated with 10 nM, 100 nM, 1 µM, or 10 µM simvastatin for 48h. **(B, C)** Cell Viability assay in MDA-MB-231 and TUBO cells treated with the indicated concentrations of Atorvastatin for 48 h. **(D, E)** Cell Viability assay in MDA-MB-231 and TUBO p140Cap cells treated with the indicated concentrations of simvastatin (SIMV) for 48 h with or without the addition of Cholesterol in the cell medium. ANOVA (*P<0.05; **P<0.01; ***P<0.001). Error bar: SEM.
