# Supplementary material for: p140Cap modulates the mevalonate pathway decreasing cell migration and enhancing drug sensitivity in breast cancer cells
Source: Cell Death Dis. 2023 Dec 20;14(12):849. doi: 10.1038/s41419-023-06357-z (PMC10733353; doi:10.1038/s41419-023-06357-z)
Supplement: Supplementary file 8 — Western Blot uncropped images [file 41419_2023_6357_MOESM8_ESM.pdf]

Original data

Western blots uncropped images

Fig 2C

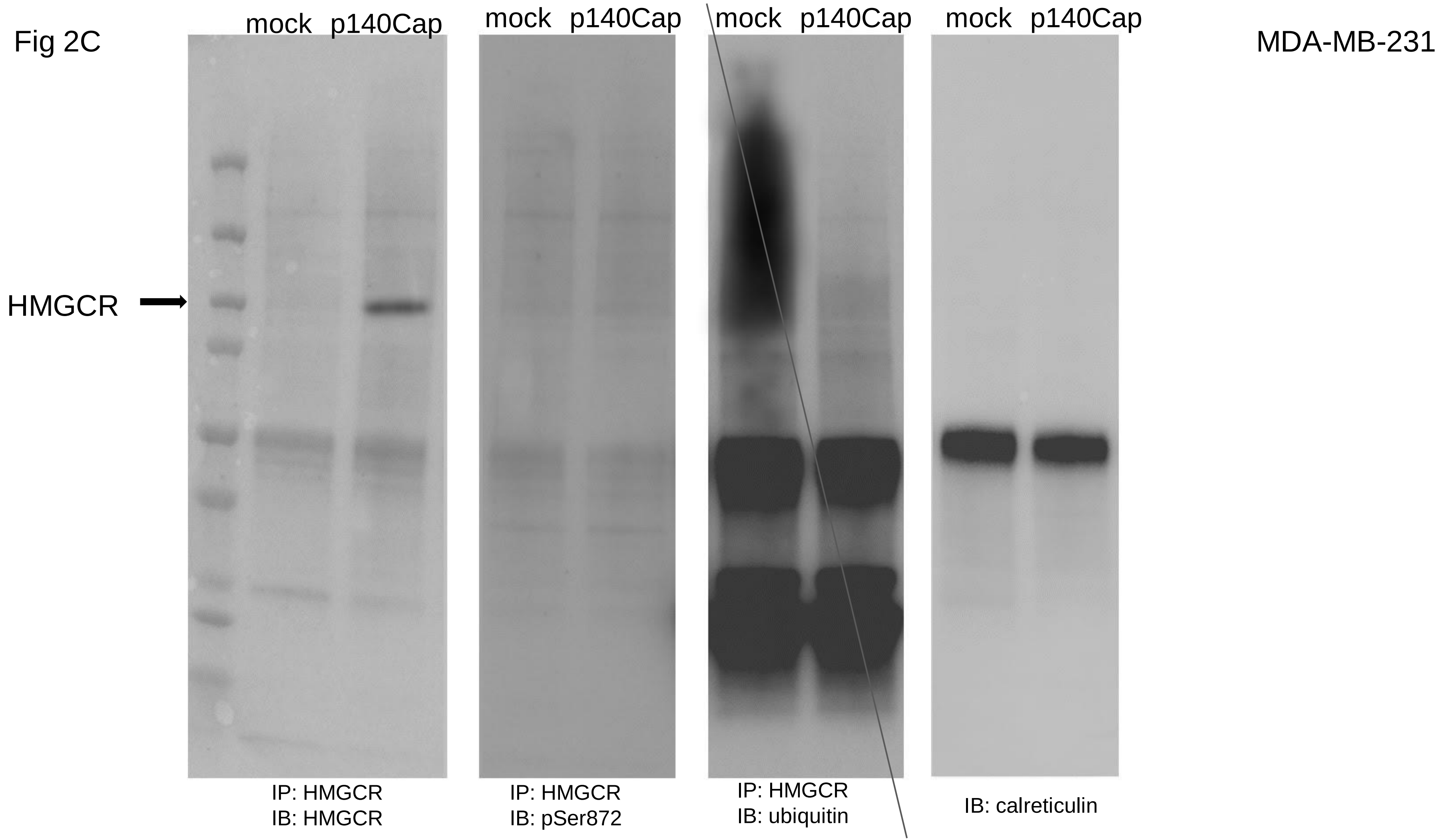
mock
p140Cap
mock
p140Cap
mock
p140Cap
mock
p140Cap
MDA-MB-231
HMGCR
IP: HMGCR
IB: HMGCR
IP: HMGCR
IB: pSer872
IP: HMGCR
IB: ubiquitin
IB: calreticulin

Fig 2C

WB anti-HMGCR e equal control (estratti microsomiali)

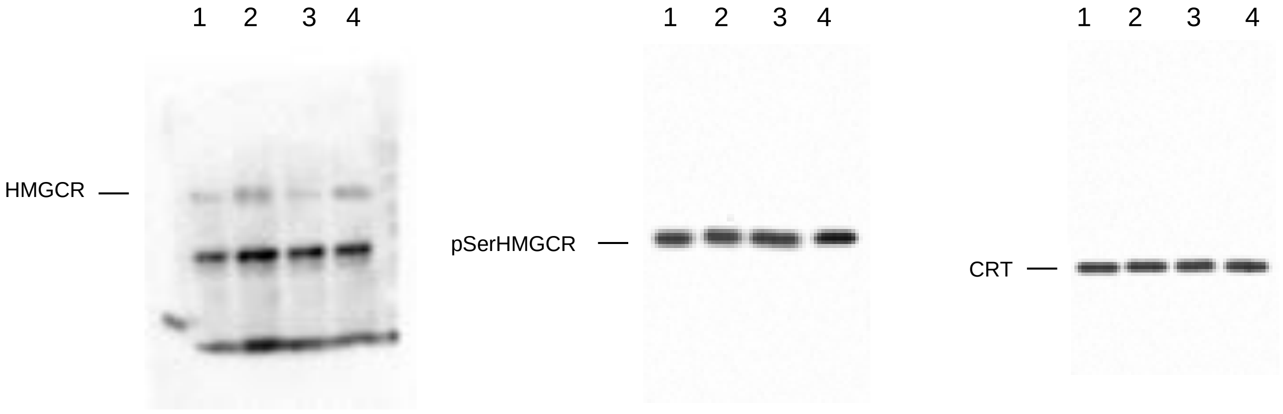

1: SKBR3 mock
2: SKBR3 p140
3: ANC mock
4: ANC p140

HMGCR in MDA cholesterol loading and depletion

Fig 2G

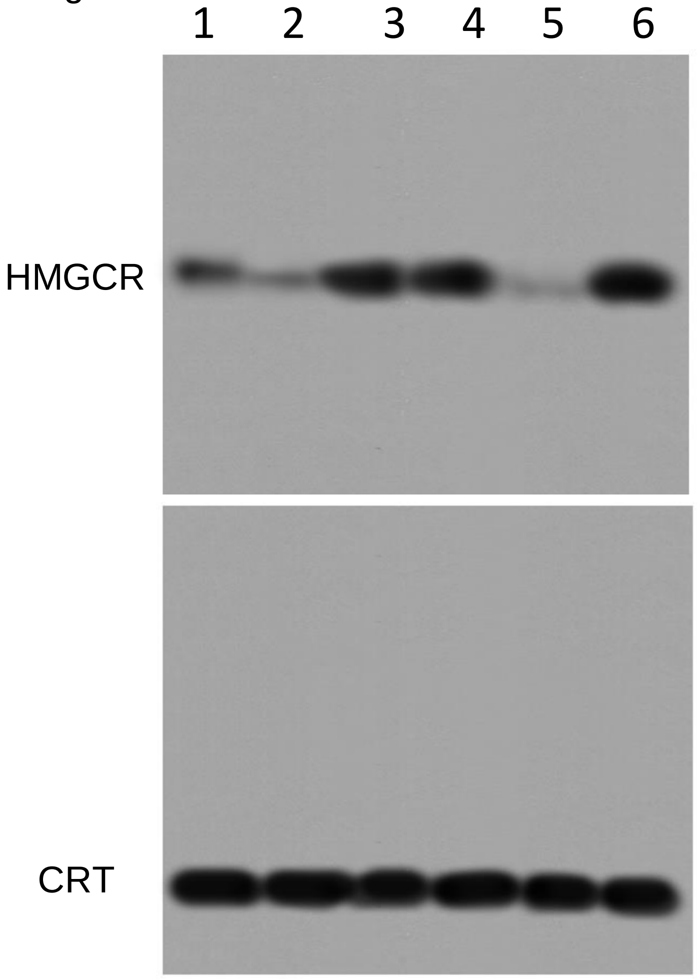

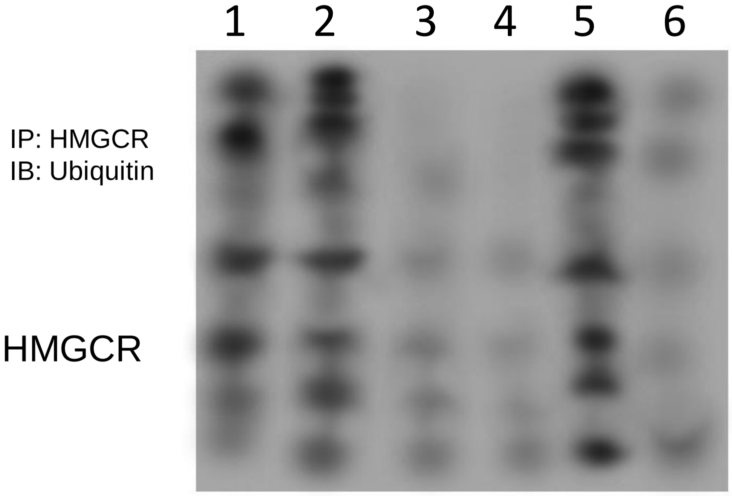

1: MDA-MB-231 mock
2: MDA-MB-231 mock, chol load 10 mM
3: MDA-MB-231 mock, chol depleted (b-methylciclodextrin)
4: MDA-MB-231 p140Cap
5: MDA-MB-231 p140Cap, chol load 10 mM
6: MDA-MB-231 p140Cap, chol depleted (b-methylciclodextrin)

Fig 2H

HMGCR in SKBR3 cholesterol loading and depletion

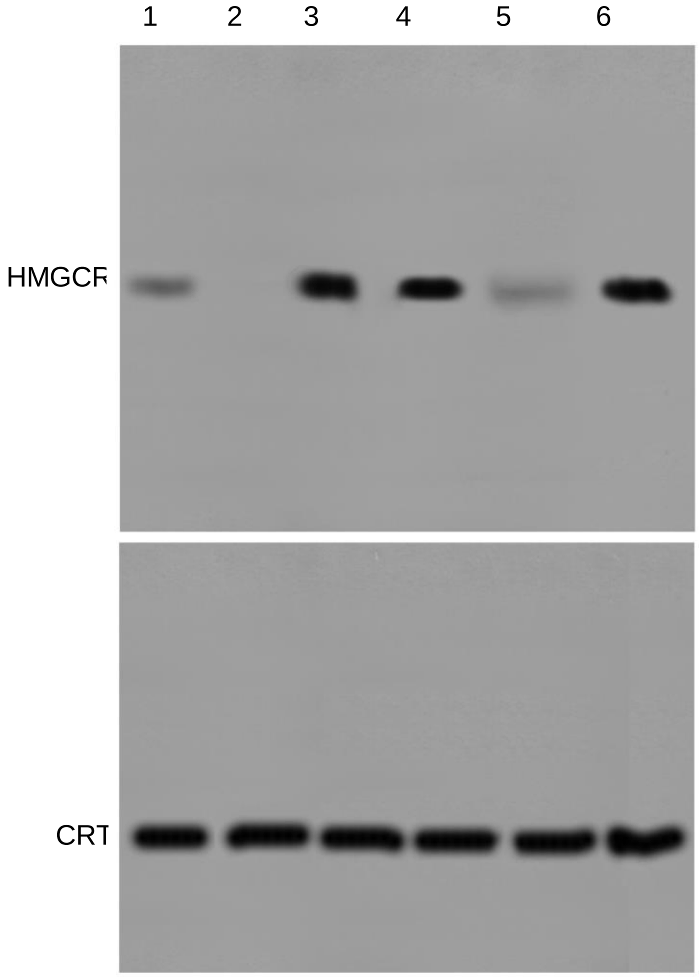

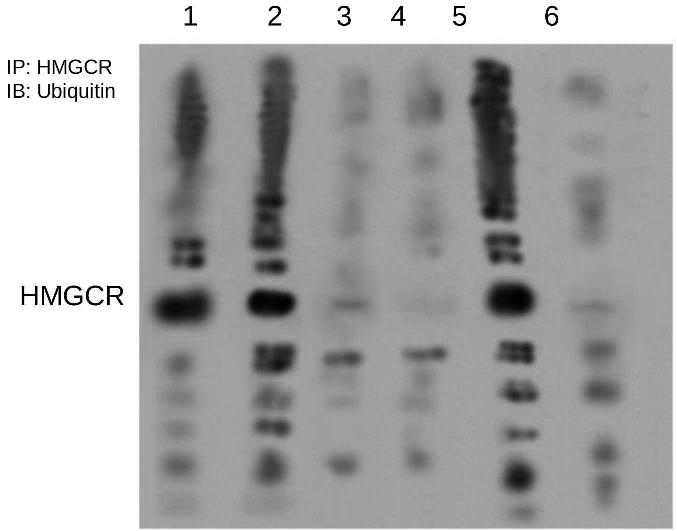

1: SKBR3 mock
2: SKBR3 mock, chol load 10 mM
3: SKBR3 mock, chol depleted (b-methylciclodextrin)
4: SKBR3 p140
5: SKBR3 p140, chol load 10 mM
6: SKBR3 p140, chol depleted (b-methylciclodextrin)

Fig 2M

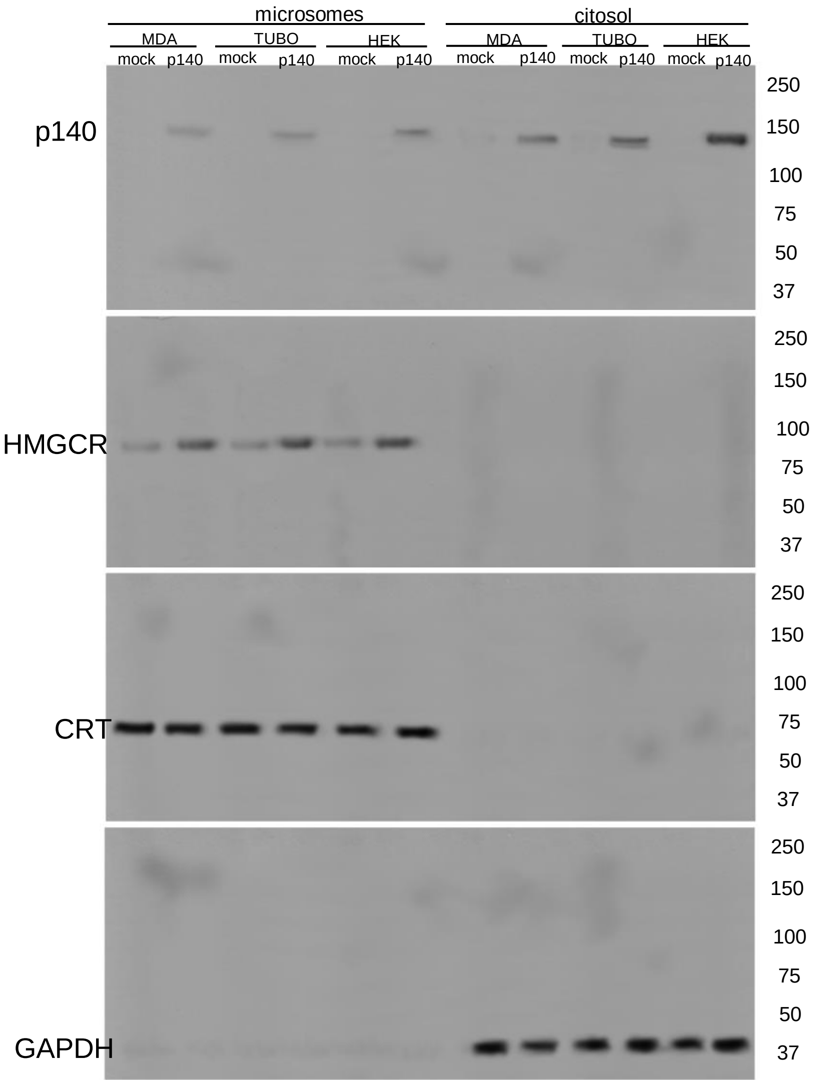
microsomes
citosol
MDA
TUBO
HEK
MDA
TUBO
HEK
mock p140 mock p140 mock p140 mock p140 mock p140 mock p140
p140
HMGCR
CRT
GAPDH
250
150
100
75
50
37

Fig 5C

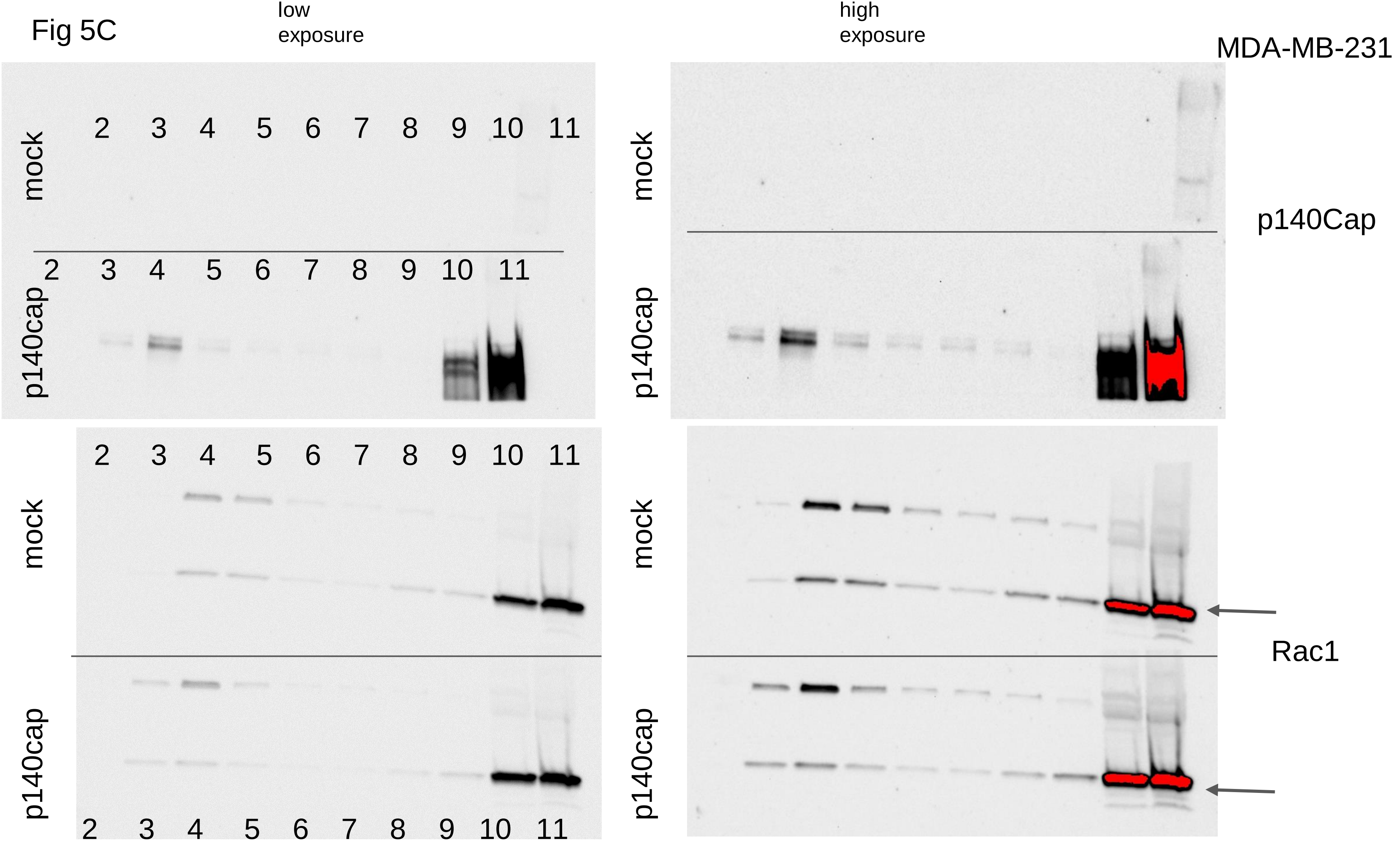
low exposure
high exposure
MDA-MB-231
mock
p140cap
2 3 4 5 6 7 8 9 10 11
p140Cap
Rac1

Fig 5C

MDA-MB-231

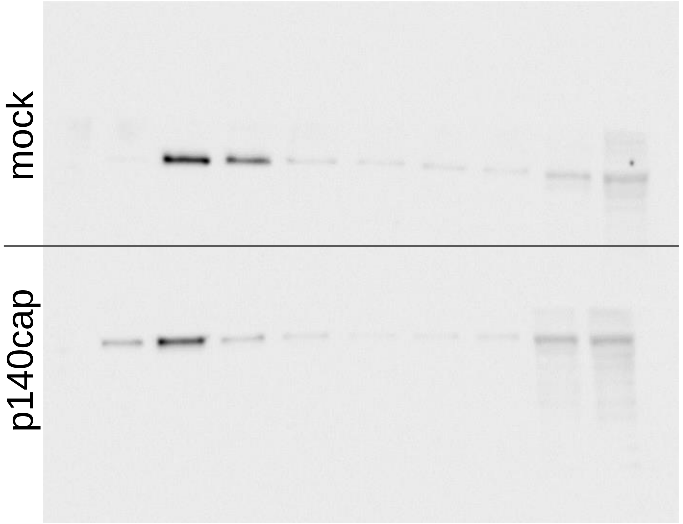

Antibody: Flotillin-1

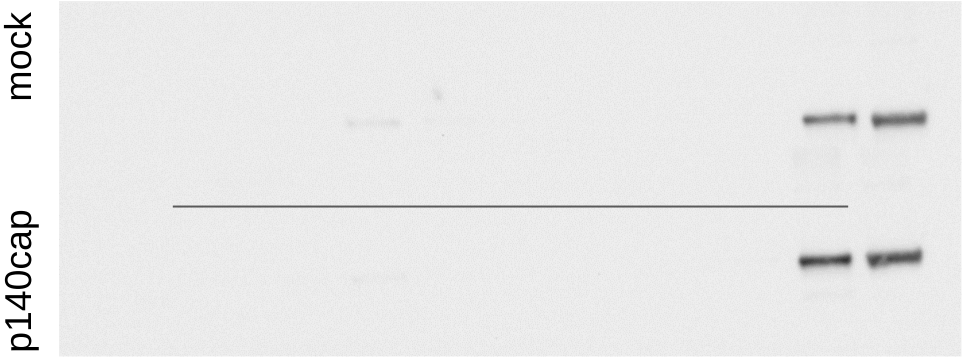

Antibody: CD71

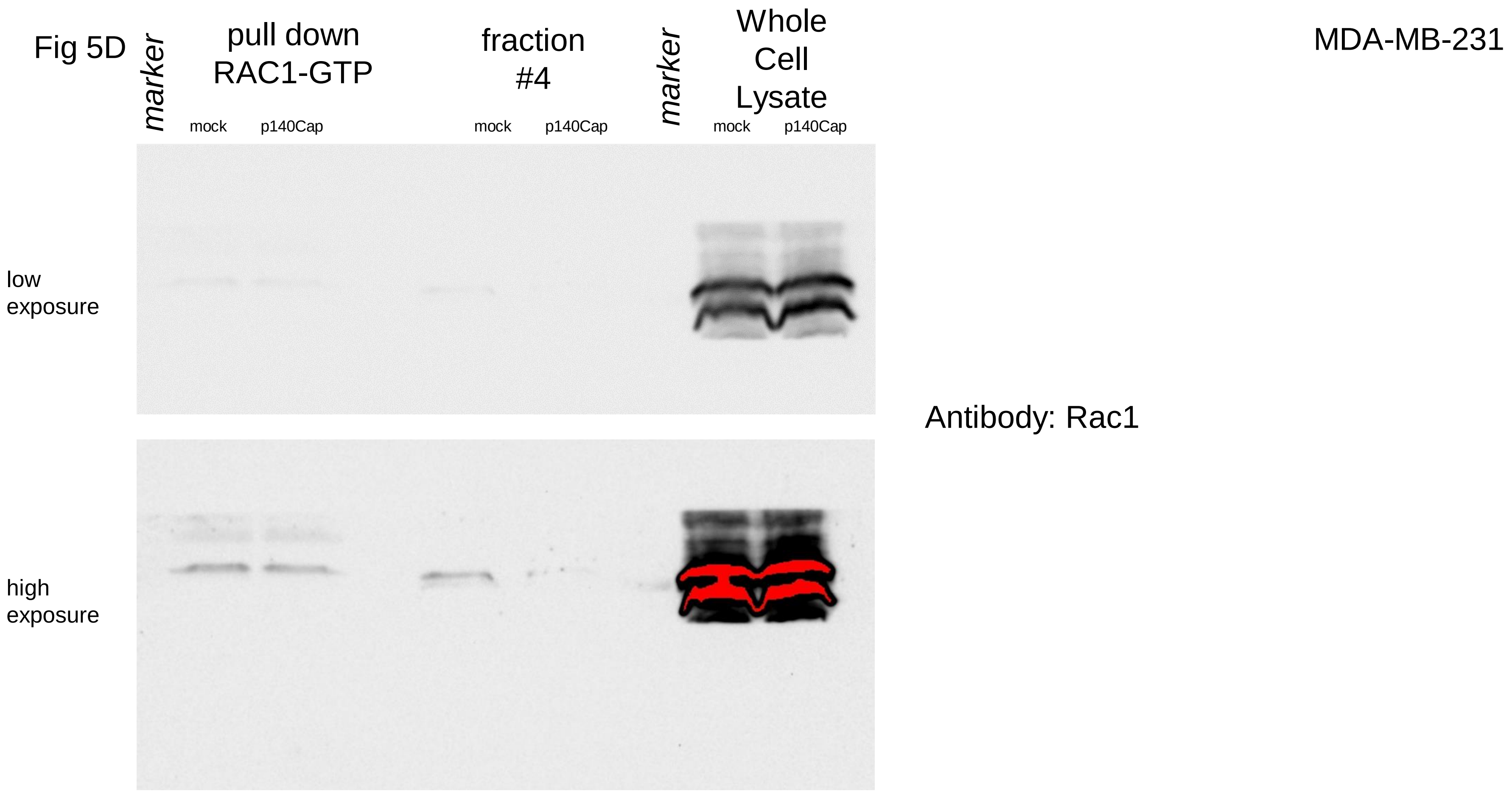
Fig 5D
marker
pull down
RAC1-GTP
fraction
#4
marker
Whole
Cell
Lysate
mock
p140Cap
mock
p140Cap
mock
p140Cap
MDA-MB-231
low
exposure
high
exposure
Antibody: Rac1

Fig 5D

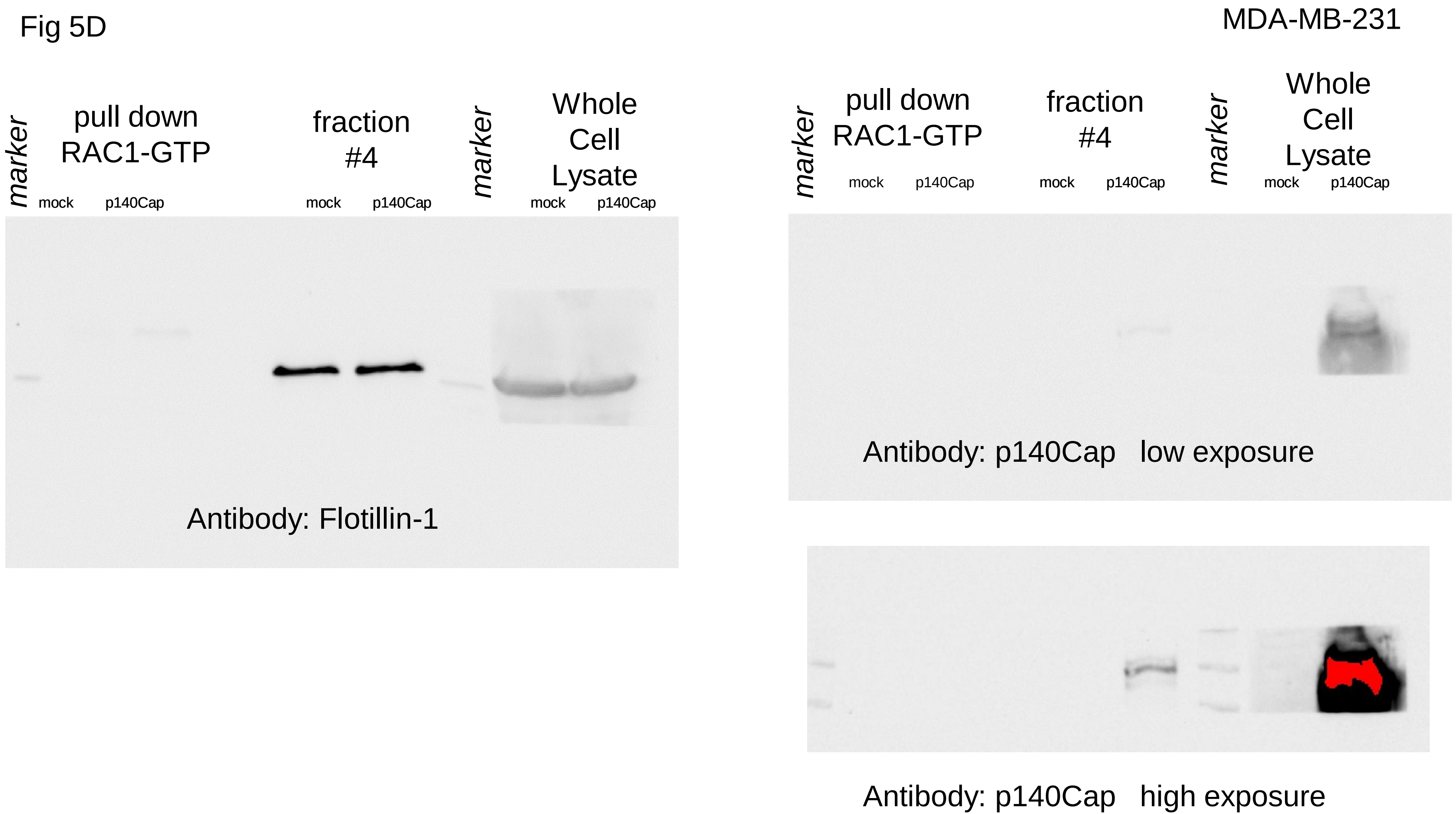
MDA-MB-231
marker
pull down RAC1-GTP
fraction #4
marker
Whole Cell Lysate
mock
p140Cap
mock
p140Cap
mock
p140Cap
Antibody: Flotillin-1
marker
pull down RAC1-GTP
fraction #4
marker
Whole Cell Lysate
mock
p140Cap
mock
p140Cap
mock
p140Cap
Antibody: p140Cap low exposure
Antibody: p140Cap high exposure

Suppl Fig 1A p140Cap cell lines

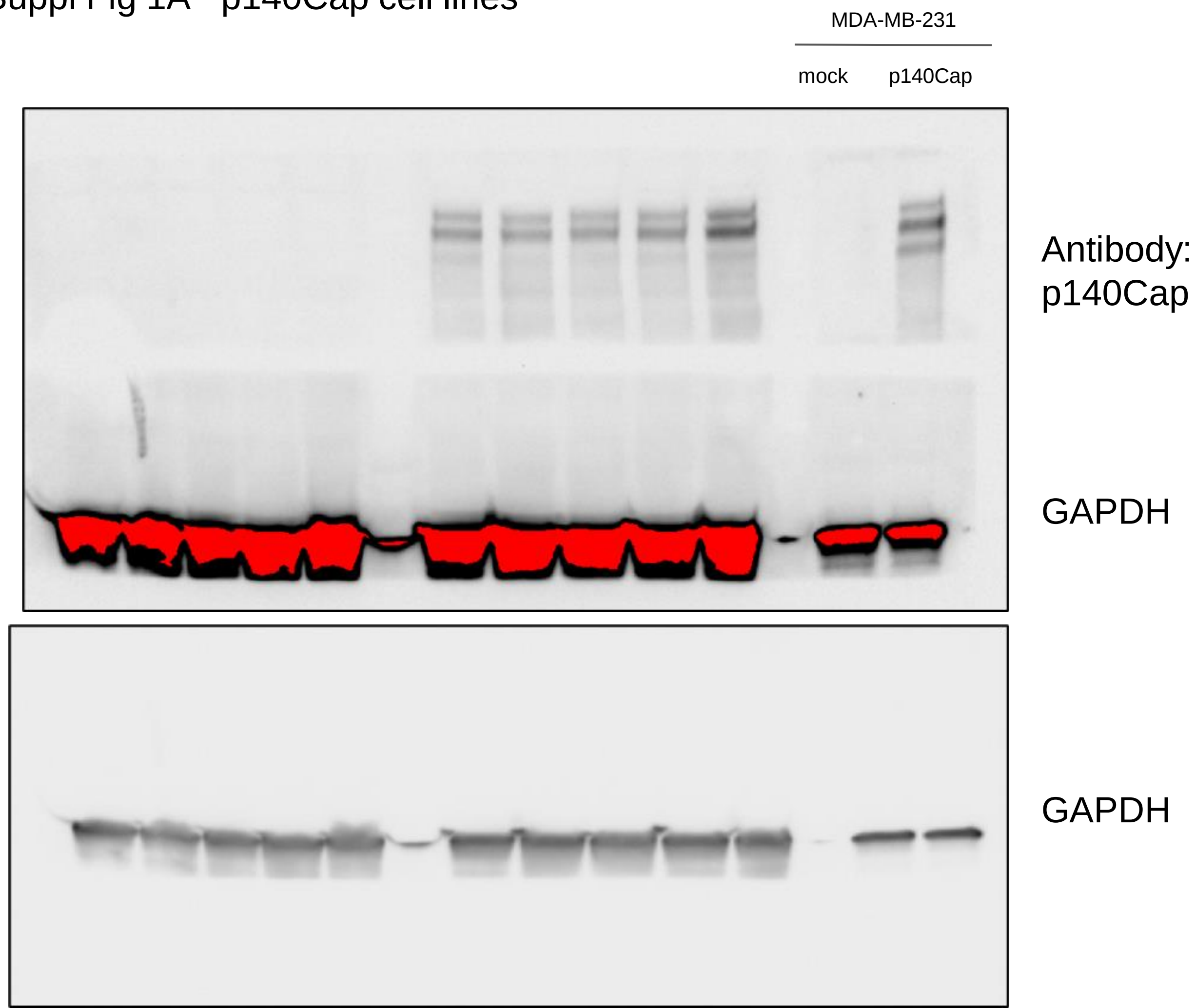

Suppl Fig 1A   p140Cap cell lines

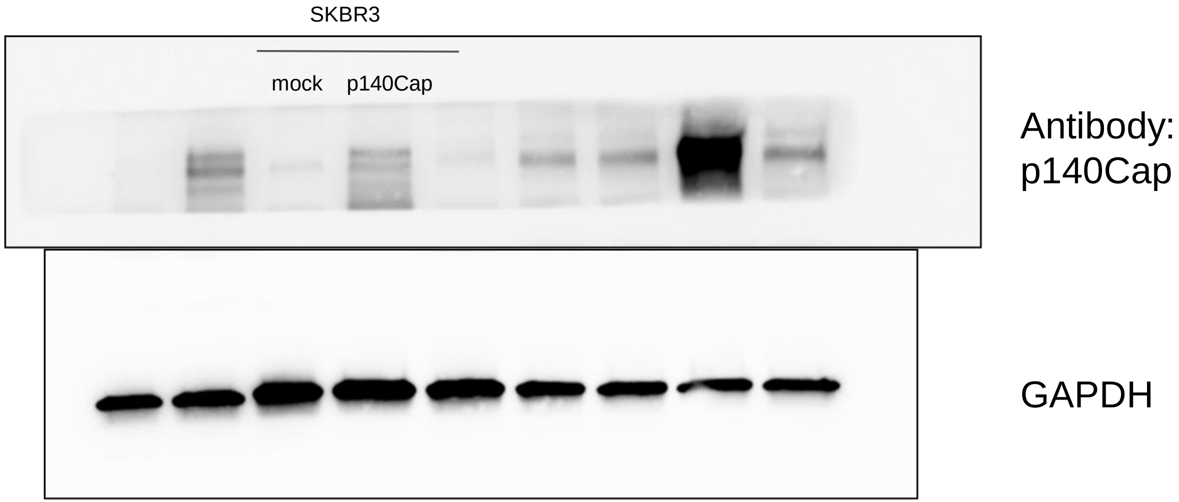

Suppl Fig 1A   p140Cap cell lines

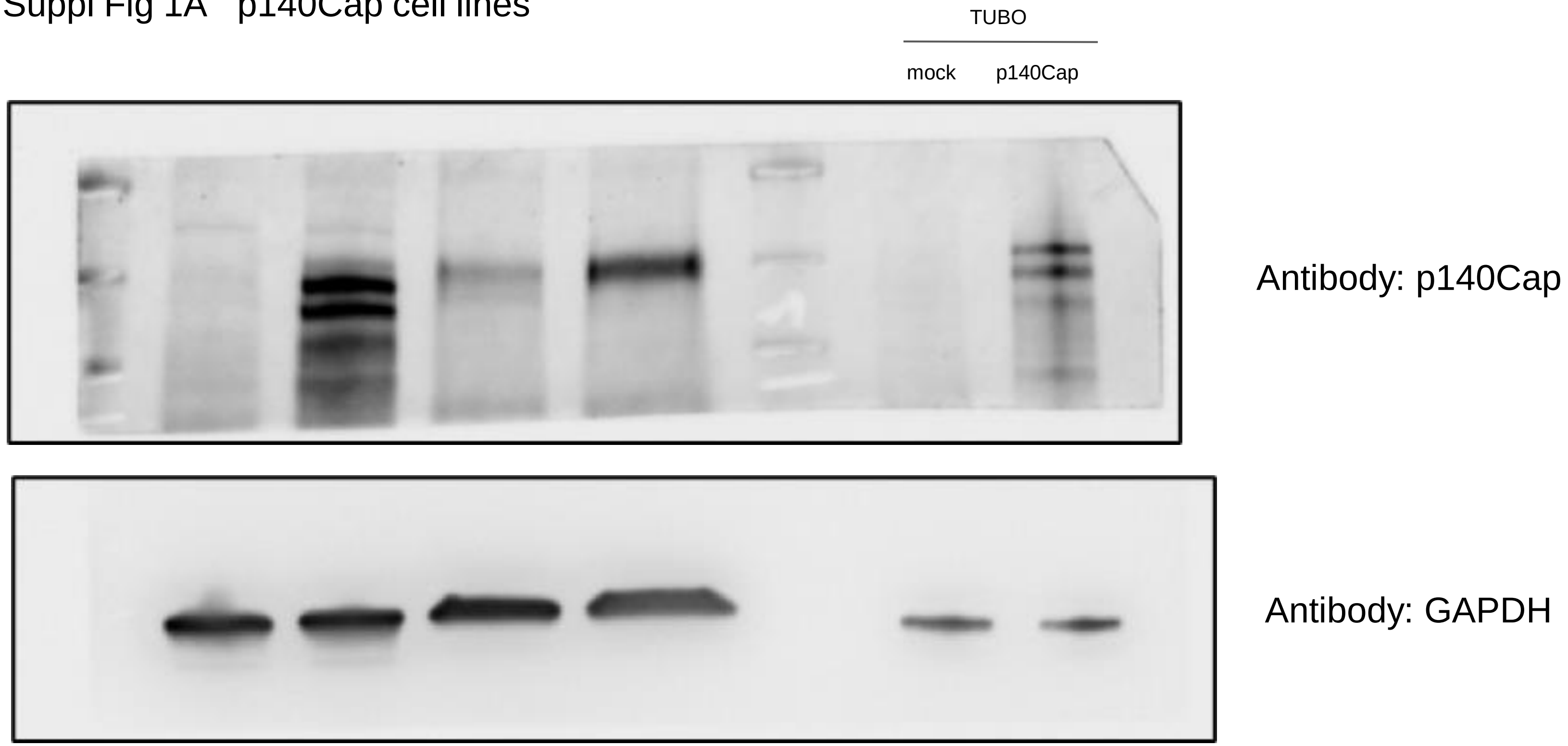

Suppl Fig 1A   p140Cap cell lines

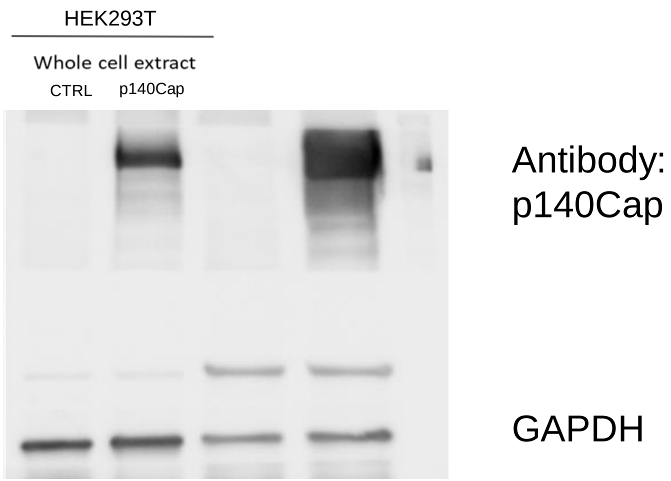

Suppl Fig 1G

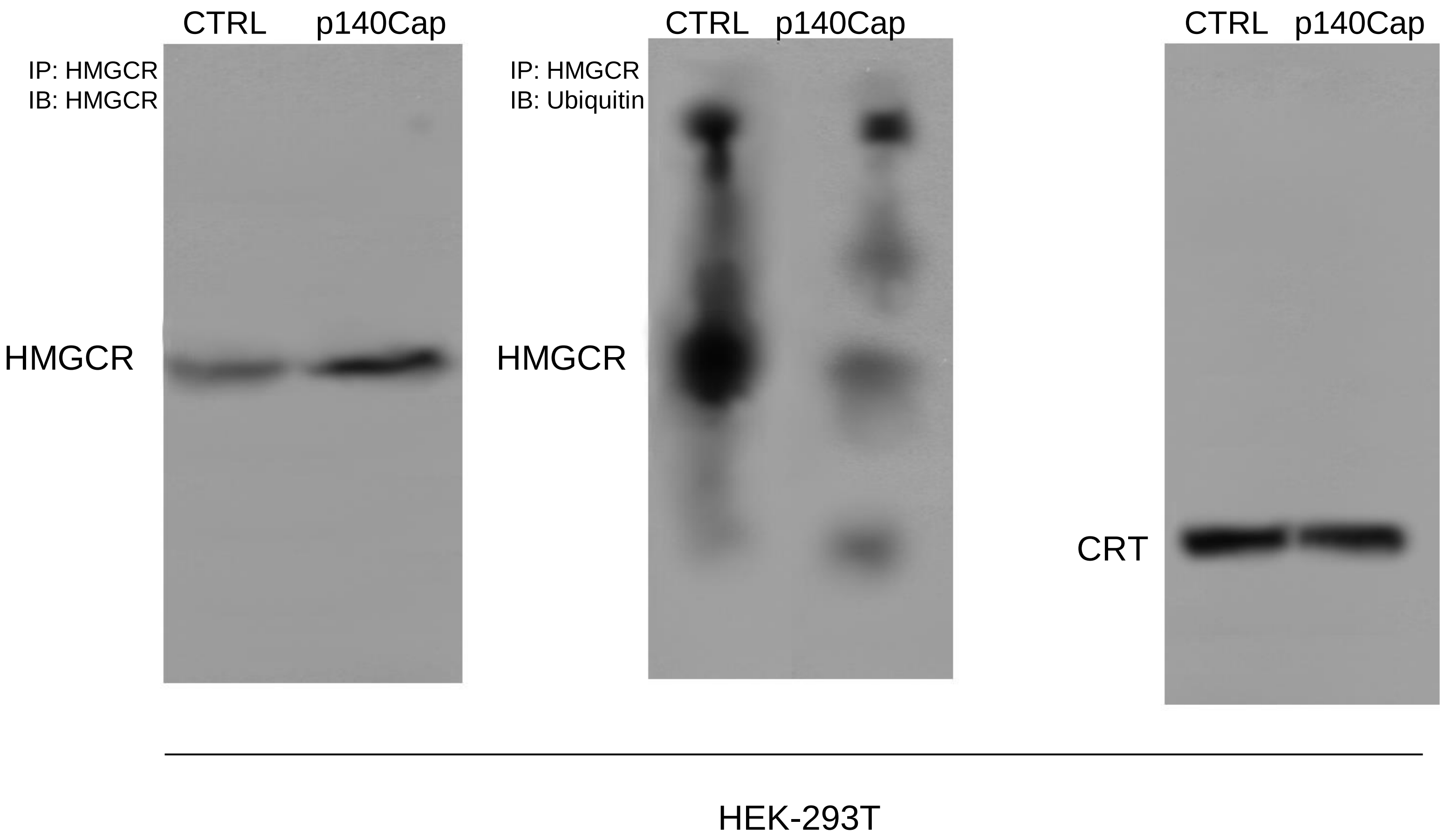
CTRL
p140Cap
IP: HMGCR
IB: HMGCR
HMGCR
CTRL
p140Cap
IP: HMGCR
IB: Ubiquitin
HMGCR
CTRL
p140Cap
CRT
HEK-293T

Suppl Fig 1H

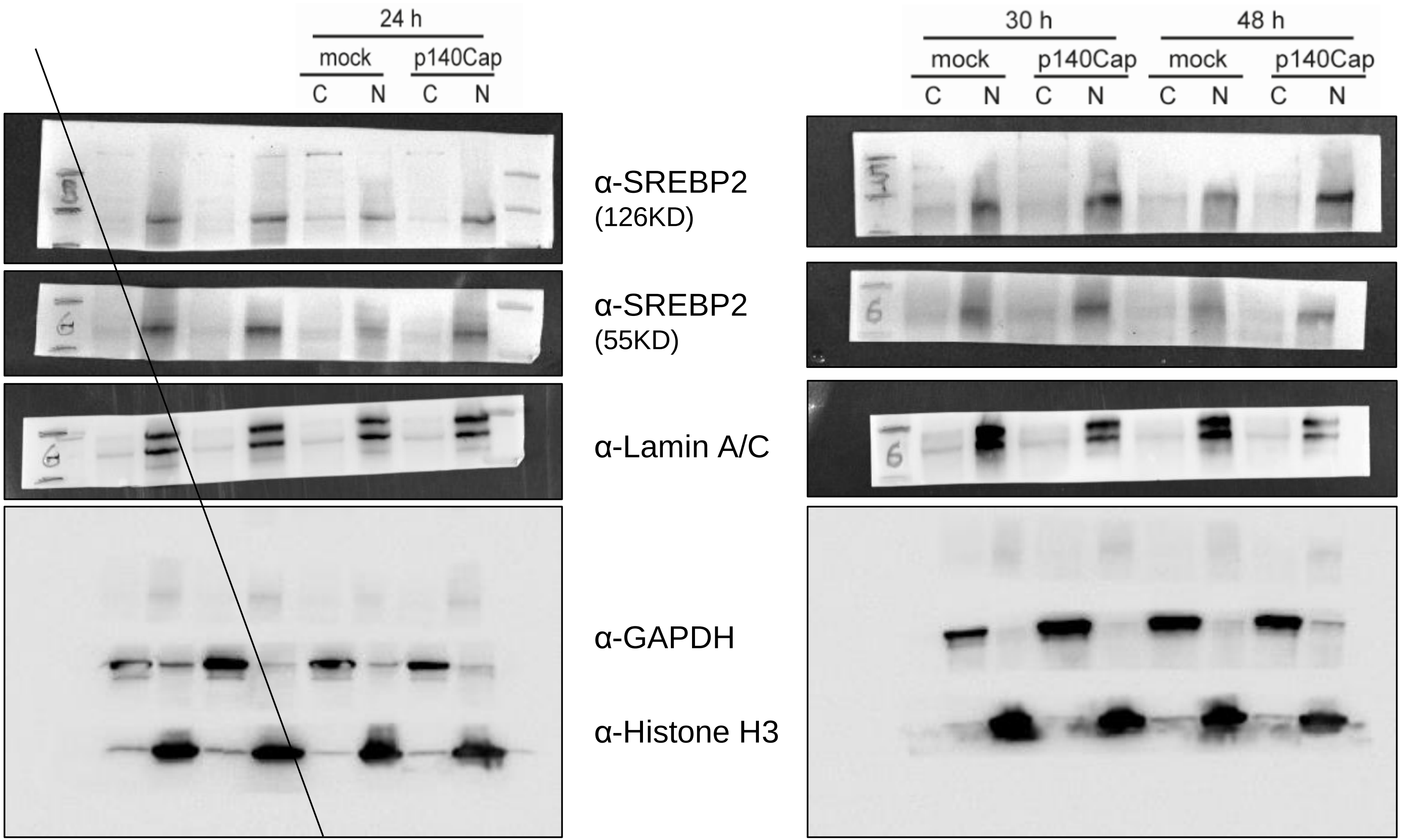

24 h
mock
p140Cap
C
N
C
N
30 h
48 h
mock
p140Cap
mock
p140Cap
C
N
C
N
C
N
C
N
α-SREBP2
(126KD)
α-SREBP2
(55KD)
α-Lamin A/C
α-GAPDH
α-Histone H3

Suppl Fig 1J

HMGCR in SKBR3 con bortezomib

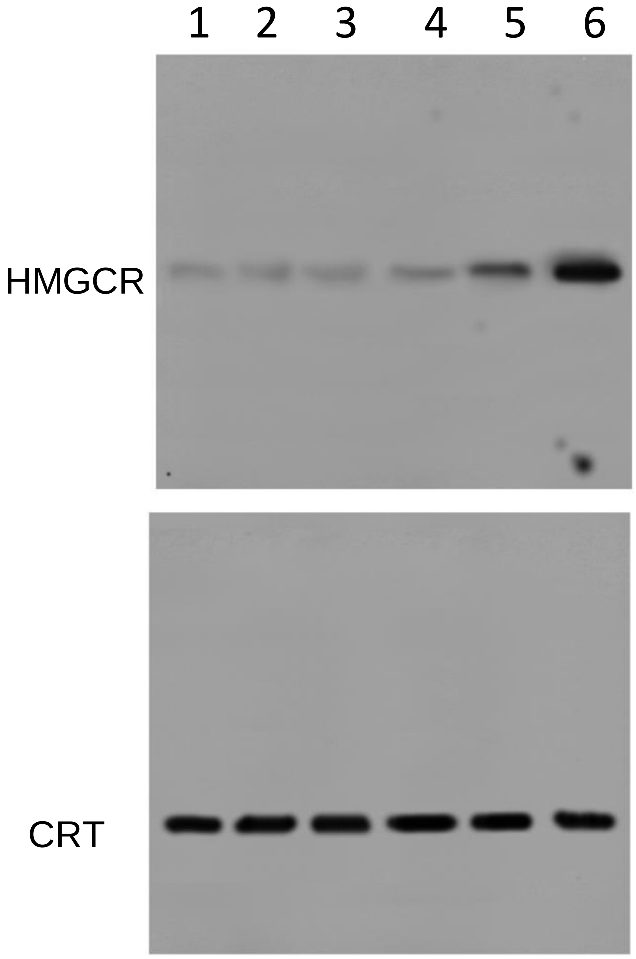

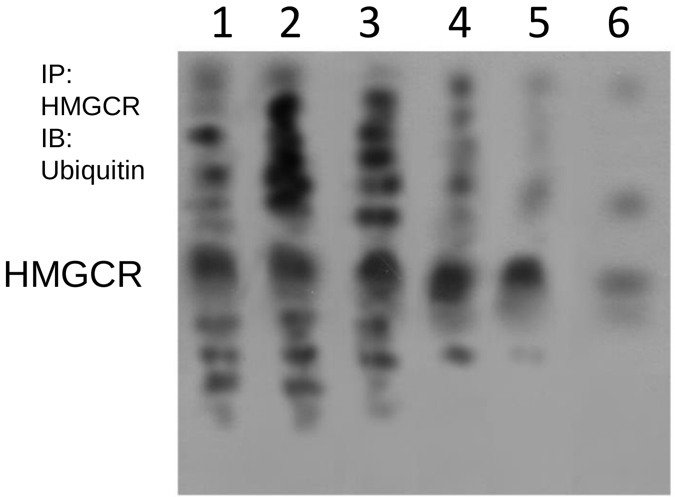

1: SKBR3 mock (viability: 100%)
2: SKBR3 mock, bortezomib 500 nM 6 h (viability: 87%)
3: SKBR3 mock, bortezomib 500 nM 3 h (viability: 91%)
4: SKBR3 mock, bortezomib 50 nM 6 h (viability: 90%)
5: SKBR3 mock, bortezomib 50 nM 3 h (viability: 97%)
6: SKBR3 p140 (viability: 94%)

Suppl Fig 1M

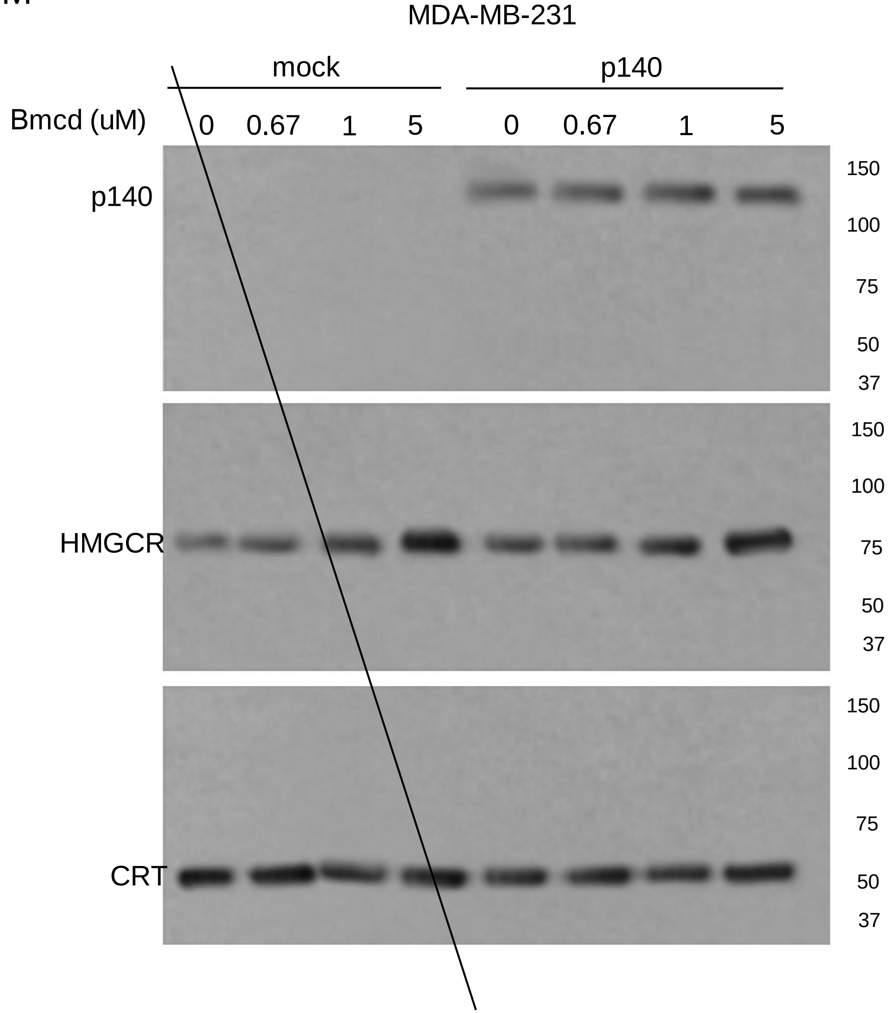
MDA-MB-231
mock
p140
Bmcd (uM)
0
0.67
1
5
0
0.67
1
5
p140
HMGCR
CRT
150
100
75
50
37
150
100
75
50
37
150
100
75
50
37
